# Development of an in vivo cleavable donor plasmid for targeted transgene integration by CRISPR-Cas9 and CRISPR-Cas12a

**DOI:** 10.1038/s41598-022-22639-6

**Published:** 2022-10-22

**Authors:** Riki Ishibashi, Ritsuko Maki, Satsuki Kitano, Hitoshi Miyachi, Fumiko Toyoshima

**Affiliations:** 1 Department of Biosystems Science, Institute for Life and Medical Sciences, Kyoto University, Sakyo-Ku, Kyoto, 606-8507 Japan; 2 Department of Mammalian Regulatory Networks, Graduate School of Biostudies, Kyoto University, Sakyo-Ku, Kyoto, 606-8502 Japan; 3 Reproductive Engineering Team, Institute for Life and Medical Sciences, Kyoto University, Sakyo-Ku, Kyoto, 606-8507 Japan

**Keywords:** Biological techniques, Biotechnology, Genetics

## Abstract

The CRISPR-Cas system is widely used for genome editing of cultured cells and organisms. The discovery of a new single RNA-guided endonuclease, CRISPR-Cas12a, in addition to the conventional CRISPR-Cas9 has broadened the number of editable target sites on the genome. Here, we developed an in vivo cleavable donor plasmid for precise targeted knock-in of external DNA by both Cas9 and Cas12a. This plasmid, named pCriMGET_9-12a (plasmid of synthetic CRISPR-coded RNA target sequence-equipped donor plasmid-mediated gene targeting via Cas9 and Cas12a), comprises the protospacer-adjacent motif sequences of Cas9 and Cas12a at the side of an off-target free synthetic CRISPR-coded RNA target sequence and a multiple cloning site for donor cassette insertion. pCriMGET_9-12a generates a linearized donor cassette in vivo by both CRISPR-Cas9 and CRISPR-Cas12a, which resulted in increased knock-in efficiency in culture cells. This method also achieved > 25% targeted knock-in of long external DNA (> 4 kb) in mice by both CRISPR-Cas9 and CRISPR-Cas12a. The pCriMGET_9-12a system expands the genomic target space for transgene knock-in and provides a versatile, low-cost, and high-performance CRISPR genome editing tool.

## Introduction

Clustered regularly interspaced short palindromic repeats (CRISPR)-CRISPR-associated (Cas) systems are adaptive immunity systems of bacteria and archaea that prevent infection by viruses and plasmids^1–3^. CRISPR-Cas systems are classified as Class 1 (Types I, III, and IV) and Class 2 (Types II, V, and VI)^4,5^. CRISPR-Cas9 is a Type II CRISPR-Cas system that has been widely used in genome editing technologies^1,6–10^. The most commonly used CRISPR-Cas9-mediated genome editing system has two main components: a Cas9 nuclease from *Streptococcus pyogenes* (SpCas9) and a guide RNA (gRNA). When recruited to a target DNA locus by gRNA, SpCas9 induces a blunted-end DNA double-strand break in a manner that is dependent on a protospacer adjacent motif (PAM) sequence (NGG; N = A/T/G/C)^11,12^.

CRISPR-Cas12a (also known as Cpf1) is a Type V CRISPR-Cas system that has been used as an alternative genome editing tool to CRISPR-Cas9^13–15^. The gRNA of the CRISPR-Cas12a ribonucleoprotein complex is a 41–44-nucleotide-long crRNA that codes a complementary sequence of 20–24 nucleotides against the target DNA sequence and does not need tracrRNA to recruit Cas12a to the target DNA locus. The commonly used *Acidaminococcus* sp. Cas12a (AsCas12a) recognizes the T-rich PAM sequence (TTTV; V = A/C/G) for induction of a DNA double-strand break^16–18^. These distinct features of CRISPR-Cas12a expand the target region for genome editing that cannot be edited by CRISPR-Cas9.

Various technologies have been developed for the CRISPR-Cas9 system to increase the targeting efficiency. Microhomology-mediated end-joining (MMEJ)^19^ and homology-independent targeted integration (HITI)^20^ methods achieved targeted transgene integration with a minimum length of homology arms. The Easi-CRISPR targeting method, which uses long single-stranded DNA (ssDNA) as a donor template, has successfully generated knock-in mice^21,22^. Another group reported the Tild-CRISPR method, in which PCR-amplified or in vitro-digested linearized double-stranded DNA is used as a donor template^23^. A homology-mediated end-joining (HMEJ)-based targeting strategy, which uses in vivo cleavable plasmid as a donor template, also exhibited high knock-in efficiency^24–26^. In addition, a donor DNA-Cas9 conjugation strategy, such as use of the Cas9-Avidin–Biotin ssDNA (CAB) system^27^ and SNAP-tag system^28^, has been reported to improve knock-in efficiency. The two-cell homologous recombination (2C-HR)-CRISPR method^29^ and adeno-associated virus (AAV)-mediated large fragment delivery strategy^30,31^ have also achieved the integration of large transgenes.

Although the CRISPR-Cas9 system has achieved targeted knock-in of long external DNA, the CRISPR-Cas12a-mediated large transgene integration has not been succeeded in mice. Previously, we developed an in vivo cleavable donor plasmid, pCriMGET (plasmid of synthetic CRISPR-coded RNA target sequence-equipped donor plasmid-mediated gene targeting), for CRISPR-Cas9 genome editing^32^. The pCriMGET system linearized a donor cassette intracellularly and enhanced the integration of long (3 kb) external DNA by CRISPR-Cas9^32^. Here, we developed the next generation of pCriMGET that enables precise knock-in of long (4.0–5.4 kb) external DNA by both CRISPR-Cas9 and CRISPR-Cas12a in culture cells and in mice.

## Results

### Development of the pCriMGET_9-12a plasmid

**Figure 1:**
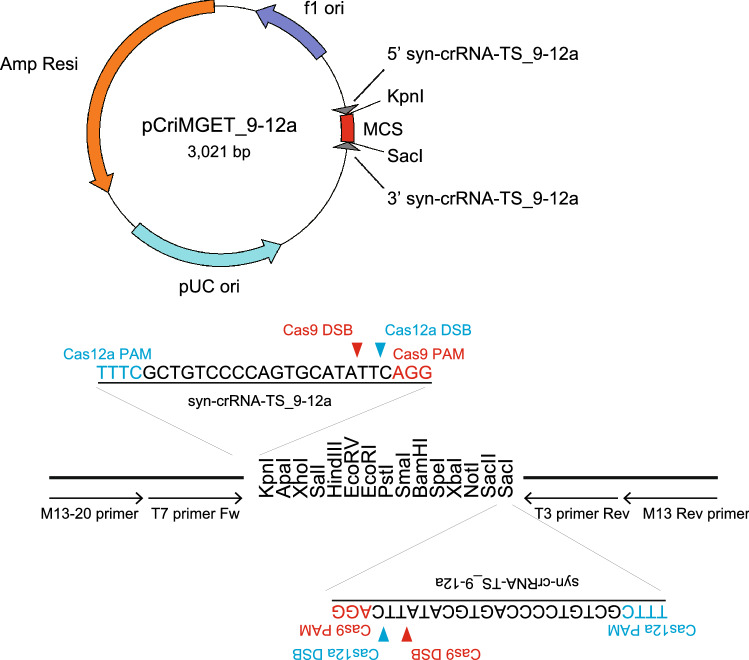
Development of the pCriMGET_9-12a plasmid. pCriMGET_9-12a harbors syn-crRNA-TS_9-12a at the 5′ and 3′ ends of the multiple cloning site (MCS). The Cas12a and Cas9 protospacer adjacent motif (PAM) sequences are shown in blue and red, respectively. The predicted double-strand break (DSB) sites induced by Cas12a and Cas9 within syn-crRNA-TS_9-12a are indicated by blue and red arrowheads, respectively. Black arrows show the locations of the M13-20, T7, T3, and M13 universal primers. Restriction enzyme (*Kpn*I and *Sca*I) recognition sites within the MCS are shown.

The first-generation pCriMGET has an off-target-free synthetic crRNA target sequence (syn-crRNA-TS) with a CRISPR-Cas9 PAM sequence at the 3′-end^32^. We modified this sequence by adding a CRISPR-Cas12a PAM sequence (TTTC) at the 5′-end (Fig. 1). The resultant syn-crRNA-TS_9-12a sequence has no potential off-target regions for CRISPR-Cas12a in the mouse or human genome that matched more than 20 out of the 23 bases of the spacer sequence (5′-GCTGTCCCCAGTGCATATTCAGG-3′) (Supplementary Table S1). Notably, the spacer sequence for CRISPR-Cas9 within the syn-crRNA-TS_9-12a (5′-GCTGTCCCCAGTGCATATTC-3′) has five potential off-target regions in the mouse genome that matched 17 out of the 20 bases, as we reported previously in syn-crRNA-TS^32^ (Supplementary Table S1). However, we have detected no indel mutations in these regions in all knock-in embryos^32^, which indicates that syn-crRNA-TS_9-12a is largely or completely free of off-target effects for both CRISPR-Cas12a and CRISPR-Cas9. We also eliminated an extra synthetic poly(A) site sequence that was outside the syn-crRNA-TS of pCriMGET, and provided a more versatile multiple cloning site (Fig. 1). This second-generation pCriMGET, which we named pCriMGET_9-12a, is expected to function as a donor plasmid that can be cleaved in vivo by both CRISPR-Cas9 and CRISPR-Cas12a.

### Precise in-frame knock-in of exogenous DNA by pCriMGET_9-12a in cultured cells

**Figure 2:**
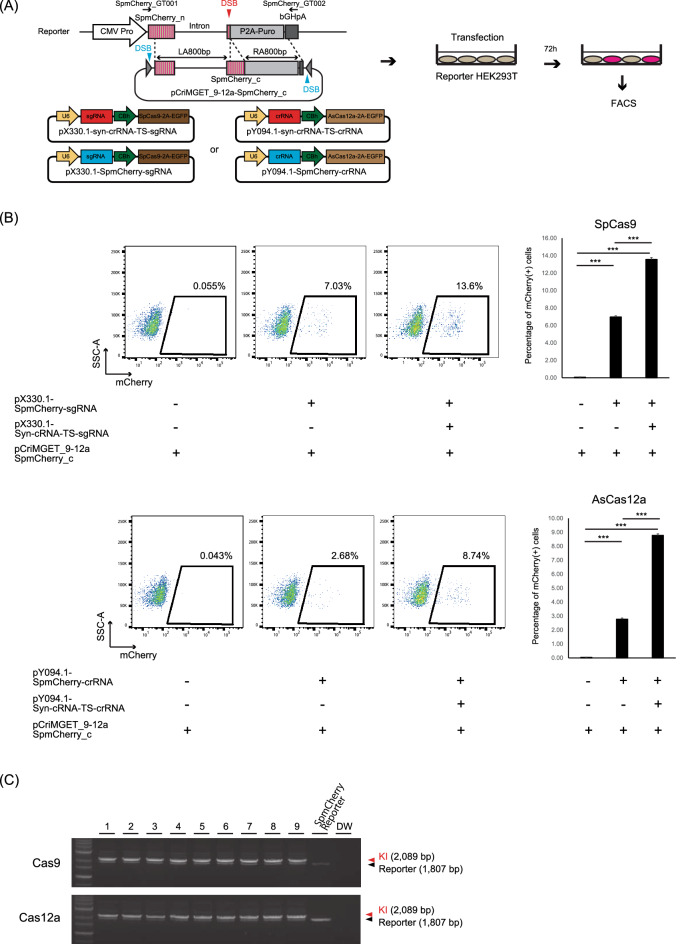
Precise in-frame knock-in of exogenous DNA by the pCriMGET_9-12a/CRISPR-Cas9 and pCriMGET_9-12a/CRISPR-Cas12a systems. (**A**) Development of the Split-mCherry reconstitution system for analyzing the in-frame knock-in efficiency of the pCriMGET_9-12a system. (**B**) Fluorescence-activated cell sorting (FACS) analysis of mCherry expression in the Split-mCherry reporter HEK293T cells transfected with the indicated plasmids (upper, pCriMGET_9-12a/CRISPR-Cas9 system; lower, pCriMGET_9-12a/CRISPR-Cas12a system). The mCherry^+^ population in EGFP^+^ cells is gated. The graphs on the right show average percentages of mCherry^+^ cells among EGFP^+^ cells in each sample. Mean ± s.d. from three experiments. ***P < 0.001, by Tukey’s multiple comparison test. (**C**) Genotyping PCRs for Split-mCherry reconstituted single-cell clones (upper, pCriMGET_9-12a/CRISPR-Cas9 system; lower, pCriMGET_9-12a/CRISPR-Cas12a system). PCR was performed by using SpmCherry_GT001 and _GT002 primer pairs, which are annealed outside of the homology arms (see (**A**)). Knock-in (KI) (2089 bp) and reporter (1807 bp) bands are indicated by red and black arrowheads, respectively.

We examined whether pCriMGET_9-12a can be applied for the in-frame knock-in of exogenous DNA by both CRISPR-Cas9 and CRISPR-Cas12a in cultured cells. To assess this, we developed the Split-mCherry (SpmCherry) reconstitution system, whereby an in-frame knock-in is monitored by mCherry expression (Fig. 2A). First, we generated a reporter HEK293T clonal cell line, in which 5′-split mCherry (SpmCherry_n-Intron-P2A-Puro) was genomically integrated (Supplementary Fig. S1). Then, we constructed a donor plasmid pCriMGET_9-12a-SpmCherry_c that had a donor cassette 3′-split mCherry (SpmCherry_c) flanked by 800-bp homology arms (Fig. 2A). We confirmed that both Cas9 and Cas12a introduce double cuts in the pCriMGET_9-12a-SpmCherry_c plasmid in vitro (Supplementary Fig. S2). The reporter HEK293T cells were transfected with pCriMGET_9-12a-SpmCherry_c with or without a pX330.1-syn-crRNA-TS-sgRNA and pX330.1-SpmCherry-sgRNA set or a pY094.1-syn-crRNA-TS-crRNA and pY094.1-SpmCherry-crRNA set for CRISPR-Cas9- and CRISPR-Cas12a-mediated genome editing, respectively. The SpCas9-2A-EGFP and AsCas12a-2A-EGFP encoded on pX330.1 and pY094.1, respectively, are expected to induce double-strand breaks at the syn-crRNA-TS of pCriMGET_9-12a-SpmCherry_c and at the genomically integrated SpmCherry site in the transfected EGFP^+^ cells (Fig. 2A). The proportion of mCherry^+^ cells in the GFP^+^ cell population was quantified by fluorescence-activated cell sorting analyses (Supplementary Fig. S3A). The mCherry^+^ cells emerged when the reporter cells were transfected with both pCriMGET_9-12a-SpmCherry_c and pX330.1-SpmCherry-sgRNA or pY094.1-SpmCherry-crRNA, but not with pCriMGET_9-12a-SpmCherry_c alone, indicating that in-frame knock-in was induced by both CRISPR-Cas9 and CRISPR-Cas12a in the cells (Fig. 2B). Moreover, co-transfection of pX330.1-syn-crRNA-TS-sgRNA or pY094.1-syn-crRNA-TS-crRNA further increased the proportion of mCherry^+^ cells, indicating that linearization of the donor cassette enhanced knock-in efficiency (Fig. 2B). Genotyping PCR of single-cell clones isolated from the mCherry^+^ cell population confirmed the targeted knock-in at the genomic level in all of the tested clones (Fig. 2C). Notably, the knock-in efficiency also depended on the length of the homology arms, with a plateau at 400–600 bp (Supplementary Fig. S3B). These findings demonstrate that the pCriMGET_9-12a/CRISPR-Cas9 and pCriMGET_9-12a/CRISPR-Cas12a systems are capable of inducing in-frame knock-in of exogenous DNA in cultured cells.

### Generation of reporter gene knock-in mice by the pCriMGET_9-12a/CRISPR-Cas9 system

**Figure 3:**
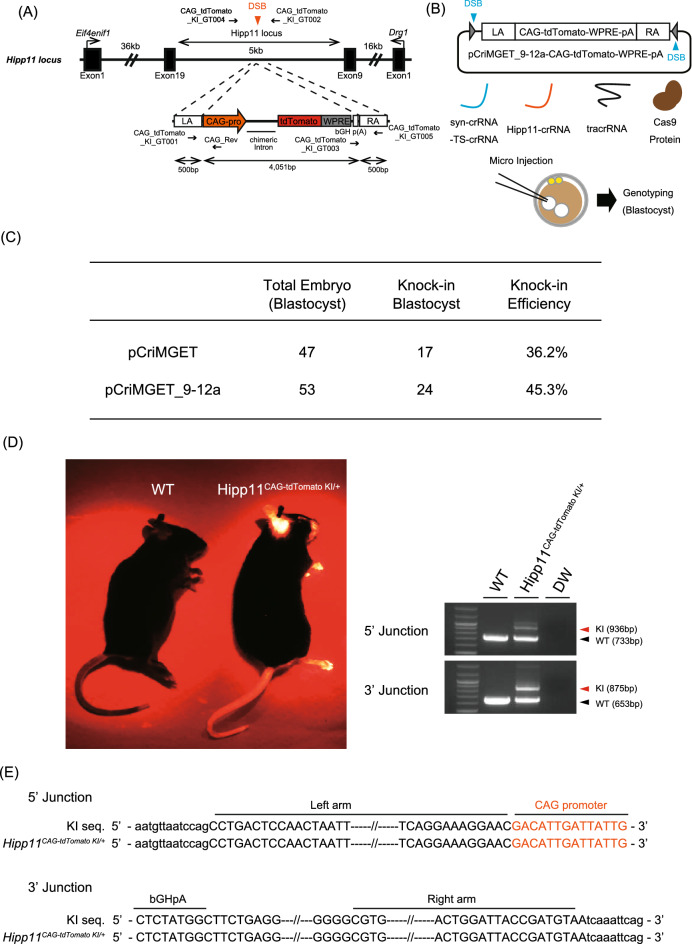
Generation of *Hipp11*^*CAG-tdTomato*^ knock-in mice by the pCriMGET_9-12a/CRISPR-Cas9 system. (**A**) pCriMGET_9-12a-mediated knock-in of the donor cassette (*CAG-tdTomato-WPRE-pA*) at the *Hipp11* locus. (**B**) Generation of *Hipp11*^*CAG-tdTomato*^ knock-in mice by the pCriMGET_9-12a/CRISPR-Cas9 system. (**C**) Knock-in frequency of pCriMGET/CRISPR-Cas9 and pCriMGET_9-12a/CRISPR-Cas9 systems in blastocysts. (**D**) Left: tdTomato expression in wild-type (WT) and *Hipp11*^*CAG-tdTomato KI/*+^ F_0_ pups. Right: Genotyping PCR for WT and *Hipp11*^*CAG-tdTomato KI/*+^ F_0_ pups (upper, 5′-junction loci; lower, 3′-junction loci). PCR was performed by using CAG-tdTomato_KI_GT001, _GT002, and _GT003 primer mixture for 3′-junction loci, and CAG-tdTomato_KI_GT004, _GT005, and CAG_Rev primer mixture for 5′-junction loci (see (**A**)). CAG_tdTomato_KI_GT002 and _GT004 primers are annealed outside of the homology arms. KI and WT bands are indicated by red and black arrowheads, respectively. (**E**) Sequence analysis of *Hipp11*^*CAG-tdTomato KI/*+^ knock-in mouse. PCR products amplified from the 5′- and 3′-junction regions from each knock-in mouse were sequenced. Upper- and lower-case letters indicate sequences inside and outside of the donor cassette, respectively.

We examined whether pCriMGET_9-12a can be applied to generate knock-in mice. We compared the knock-in frequency of the pCriMGET_9-12a/CRISPR-Cas9 system with that of the first-generation pCriMGET/CRISPR-Cas9 system. To this end, we designed a strategy for targeting knock-in of the *tdTomato* reporter gene into the *Hipp11* safe harbor locus on the mouse genome (Fig. 3A). We constructed the donor plasmid pCriMGET_9-12a-CAG-tdTomato-woodchuck hepatitis virus post-transcriptional regulatory element (WPRE)-poly(A), which incorporates the *tdTomato* gene downstream of the CAG promoter flanked by 500-bp homology arms. The donor plasmid was microinjected into the pronuclei of pronuclear-stage mouse embryo together with syn-crRNA-TS-crRNA, Hipp11-crRNA, tracrRNA, and Cas9 protein (Fig. 3B). After microinjection, the blastocysts were collected and PCR genotyped at 5′ and 3′ junction loci (Supplementary Fig. S4). The results show that 17 of the 47 injected blastocysts (36.2%) were knocked in by pCriMGET/CRISPR-Cas9 (Fig. 3C, Supplementary Fig. S4A), and 24 of the 53 injected blastocysts (45.3%) were knocked in by pCriMGET_9-12a/CRISPR-Cas9 (Fig. 3C, Supplementary Fig. S4B). We confirmed the tdTomato reporter gene expression in multiple tissues of the F_0_ pups generated by pCriMGET_9-12a/CRISPR-Cas9 (Fig. 3D, Supplementary Fig. S5). No indels or frame-shifts were detected in the 5′ and 3′ junction regions of the knock-in pups (Fig. 3E). The knock-in frequency was higher with syn-crRNA-TS-crRNA (50%) than it was without syn-crRNA-TS-crRNA (6.5%), indicating that linearization of the donor cassette enhanced the knock-in efficiency of pCriMGET_9-12a/CRISPR-Cas9 (Supplementary Fig. S6). These findings demonstrate that the pCriMGET_9-12a/CRISPR-Cas9 system induced precise knock-in of exogenous DNA at an efficiency that was comparable to that of the pCriMGET/CRISPR-Cas9 system.

### Generation of reporter gene knock-in mice by the pCriMGET_9-12a/CRISPR-Cas12a system

**Figure 4:**
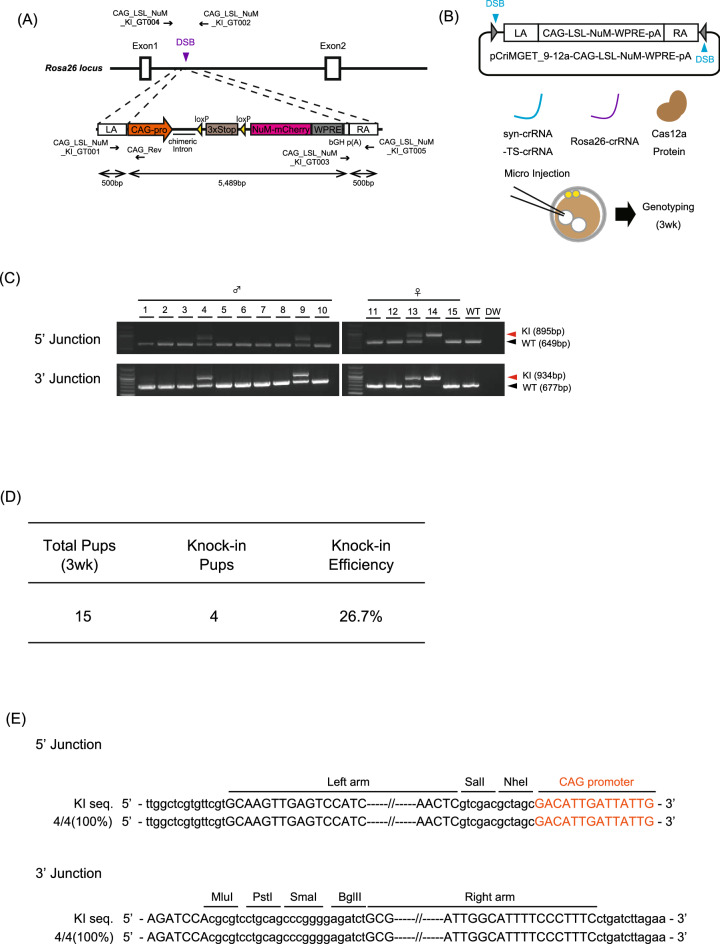
Generation of *Rosa26*^*CAG-LSL-NuM-mCherry*^ knock-in mice by the pCriMGET_9-12a/CRISPR-Cas12a system. (**A**) pCriMGET_9-12a/CRISPR-Cas12a-mediated knock-in of the donor cassette (*CAG-LSL-NuM-mCherry-WPRE-pA*) at the *Rosa26* locus. (**B**) Strategy used to generate the *Rosa26*^*CAG-LSL-NuM-mCherry*^ knock-in mice. (**C**) Genotyping PCR for *Rosa26*^*CAG-LSL-NuM-mCherry*^ knock-in F_0_ pups at 3 weeks old. PCR was performed using Rosa26-LSL-NuM_KI_GT001, _GT002, and _GT003 primer mixture for 3′-junction loci, and Rosa26-LSL-NuM_KI _GT004, _GT005, and CAG_Rev primer mixture for 5′-junction loci (see (**A**)). Rosa26-LSL-NuM_KI_GT002 and _GT004 primers are annealed outside of the homology arms. KI and WT bands are indicated by red and black arrowheads, respectively. (**D**) Knock-in frequency of F_0_ pups at 3 weeks old. (**E**) Sequence analysis of *Rosa26*^*CAG-LSL-NuM-mCherry*^ knock-in mice. PCR products amplified from 5′- and 3′-junction regions from each knock-in mouse were sequenced. Upper- and lower-case letters indicate sequences inside and outside of the donor cassette, respectively.

**Figure 5:**
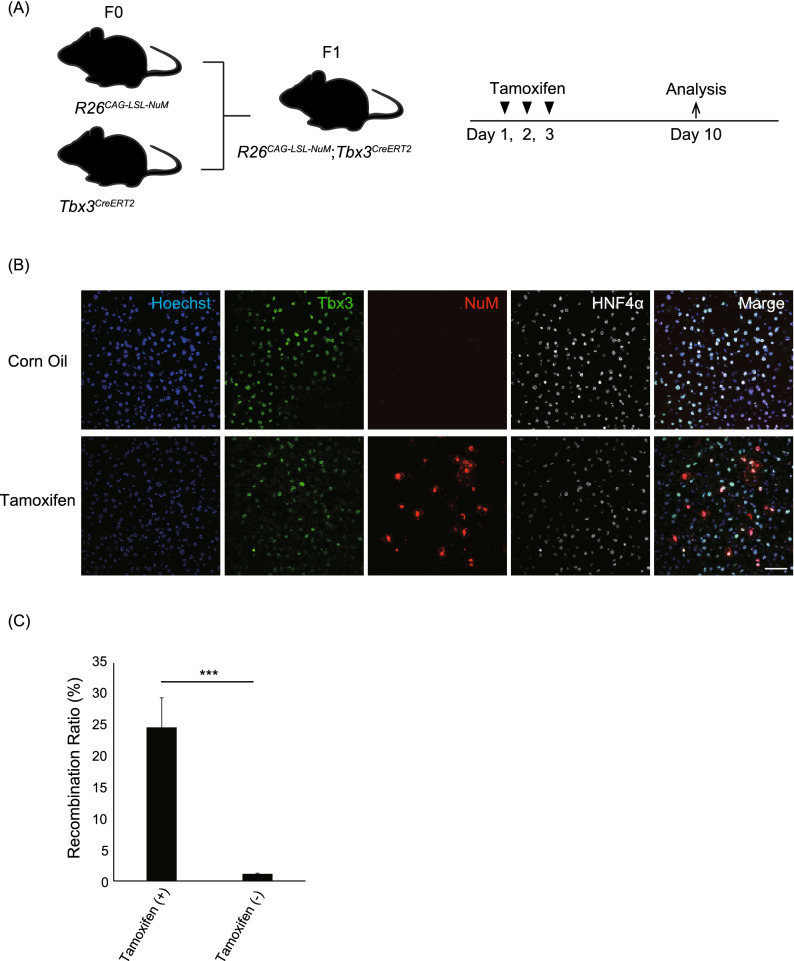
Donor transgene expression in *Rosa26*^*CAG-LSL-NuM-mCherry*^ knock-in mice. (**A**) Induction of NuM-mCherry proteins under the control of *Tbx3* gene expression. *Rosa26*^*CAG-LSL-NuM-mCherry/*+^ mice were crossed with *Tbx3*^*CreERT2/*+^ mice to generate *Rosa26*^*CAG-LSL-NuM-mCherry*^;*Tbx3*^*CreERT2*^ hemizygous mice. Intraperitoneal administration of tamoxifen into mice induced NuM-mCherry expression under the control of CreERT2 in the Tbx3^+^ cells. (**B**) Representative immunofluorescence images of liver tissue in the *Rosa26*^*CAG-LSL-NuM-mCherry*^;*Tbx3*^*CreERT2*^ mice administered control corn oil or tamoxifen. Tbx3 (green), NuM-mCherry (red), HNF4α (white), and DAPI (blue). Scale bar, 20 µm. (**C**) Recombination ratio in the control and tamoxifen-administered *Rosa26*^*CAG-LSL-NuM-mCherry*^;*Tbx3*^*CreERT2*^ mouse livers. The recombination ratio was calculated by the percentage of NuM-mCherry^+^ cells among Tbx3^+^HNF4α^+^ cells (n > 300 cells from three individual slides). Mean ± s.d. from three individual slides. ***P < 0.01, by two-tailed Student’s t test.

We assessed whether pCriMGET_9-12a/CRISPR-Cas12a can be used to generate knock-in mice. We designed a strategy for targeting knock-in of the *loxP-3* × *Stop-loxP-nuclear and membrane-mCherry* (*LSL-NuM-mCherry*) reporter gene into the *Rosa26* safe harbor locus on the mouse genome (Fig. 4A). We constructed the donor plasmid pCriMGET_9-12a-CAG-LSL-NuM-mCherry-WPRE-poly(A), which incorporates the *LSL-NuM-mCherry* gene downstream of the CAG promoter flanked by 500-bp homology arms (Fig. 4A). The donor plasmid was microinjected into the pronuclei of pronuclear-stage mouse embryo together with syn-crRNA-TS-crRNA, Rosa26-crRNA, and Cas12a protein (Fig. 4B). After transplantation into pseudopregnant mice, 3-week-old male and female mice were collected and PCR genotyped at 5′ and 3′ junction loci (Fig. 4C). The results showed that 4 of the 15 pups (26.7%) had been knocked in (Fig. 4C,D). No indels or frame-shifts were detected in the 5′ and 3′ junction regions of the knock-in pups (Fig. 4E). The knock-in frequency was higher in the presence of syn-crRNA-TS-crRNA (17.7%) than it was in its absence (7.7%), indicating that linearization of the donor cassette enhanced the knock-in efficiency of pCriMGET_9-12a/CRISPR-Cas12a (Supplementary Fig. S7A,B). Finally, we evaluated the functionality of the reporter gene in the knock-in mice. To this end, a *Rosa26*^*CAG-LSL-NuM/*+^ F_0_ pup (#9, Fig. 4C) was crossed with *Tbx3*^*creERT2*^ to obtain *Rosa26*^*CAG-LSL-NuM/*+^;*Tbx3*^*creERT2/*+^ F_1_ pups in which nuclear and membrane-mCherry signals were expected to be detected in Tbx3-expressing cells upon tamoxifen treatment (Fig. 5A, Supplementary Fig. S8). We administered tamoxifen to the F_1_ pups and dissected the liver at day 10 (Fig. 5A). Endogenous Tbx3 was detected in the hepatocytes (HNF4a^+^ cells), as reported previously^33,34^ (Fig. 5B, Tbx3). We found that > 20% of the Tbx3^+^/HNF4a^+^ hepatocytes had positive mCherry signals at the nucleus and cell cortex in the tamoxifen-administered mice, but the mCherry signal was barely detected in the control vehicle-injected mice (Fig. 5B,C). Similarly, nuclear and membrane mCherry signals were detected in plantar skin epidermis where Tbx3 is expressed^35^ (Supplementary Fig. S9). These results confirm the functionality of the transgene products in the knock-in mice, and demonstrate precise targeted integration of long (> 5 kb) external DNA by the pCriMGET_9-12a/CRISPR-Cas12a system in mice.

## Discussion

CRISPR-Cas12a has been shown to achieve gene knockout in mice and gene targeting in plants, bacteria, and mammalian cells^36–40^. Although CRISPR-Cas12a also accomplished single-base editing in mice using short (180 bases) single-stranded oligoDNA as a donor^41,42^, thus far, no study has reported the generation of transgene knock-in mice by CRISPR-Cas12a. We have shown that pCriMGET_9-12a overcomes the donor size limitation for transgene knock-in by CRISPR-Cas12a, and successfully integrated 5.4-kb-long exogenous DNA into a targeted genomic locus. Furthermore, pCriMGET_9-12a surpassed the donor size (4.0 kb) of the first-generation pCriMGET (3.0–3.3 kb)^32^ for CRISPR-Cas9-mediated transgene knock-in. This donor size is comparable to that of previously developed technologies for the integration of large transgenes, including the Cas9-Avidin/Biotin-donor DNA system^27^, SNAP tag system^28^, (2C-HR)-CRISPR method^29^, and AAV-mediated donor gene infection^30,31^. Although the transgene knock-in efficiency of pCriMGET_9-12a/CRISPR-Cas12a (17–26.7%) was lower than that of pCriMGET_9-12a/CRISPR-Cas9 (45.3–50%), the development of a modified Cas12a protein with enhanced function in future studies may improve the knock-in efficiency of this system, as was the case for Cas9^43,44^.

The HMEJ-based targeting strategy uses a single guide RNA targeting endogenous genomic locus and donor plasmid at the same time to simplify the system^24–26^. The pCriMGET_9-12a system requires two guide RNAs, namely, for the endogenous genomic target site and for the donor plasmid at syn-crRNA-TS-crRNA. Although two guide RNAs, rather than the single guide RNA, might increase the off-target risk, syn-crRNA-TS-crRNA has been confirmed to be largely or completely free of off-target effects. Because pCriMGET_9-12a is equipped with a multiple cloning site, a transgene donor cassette can be simply incorporated into the plasmid with no need to add guide RNA sequences at both ends of the donor cassette.

The first-generation pCriMGET system exhibited mosaicism, which is a common problem when generating mutant animals using CRISPR-Cas systems^32^. Mosaicism presumably also occurred in the pCriMGET_9-12a systems. However, we confirmed that the donor gene was inherited by the next generation in accordance with Mendel’s law (Supplementary Fig. S8), indicating that germinal transmission of the donor gene was achieved. Successful germline transmission can also overcome random integration in mice; for example, backcrossing the mutant mice to an inbred control strain will eliminate the randomly integrated transgene cassette from the genome. A recent study showed that fusion of the Cas9 protein with a transcription factor reduced the frequency of random integration^45^. Application of the modified Cas9 or modified Cas12a to the pCriMGET_9-12a system will help to improve this transgene knock-in methodology. Random integration is a detrimental issue in the genome editing of culture cells. Development of technology that circumvents undesirable nontargeted transgene integration in culture cells is a remaining challenge for cell-based regenerative medicine and gene therapy.

## Methods

### pCriMGET_9-12a

Mismatches of the syn-crRNA-TS_9-12a spacer sequence for CRISPR-Cas12a (5′-GCTGTCCCCAGTGCATATTCAGG-3′) and for CRISPR-Cas9 (5′-GCTGTCCCCAGTGCATATTC-3′) in the mouse (GRCm38/m10) and human (GRCh38/hg38) genomes were checked using Cas-OFFinder^46^ and CRISPOR^47^. Synthesized oligoDNAs (Thermo Fisher Scientific) were annealed and inserted into *Kpn*I and *Sca*I sites in the pBluescript II SK(+) plasmid. The insertions were confirmed by Sanger sequencing.

### pcDNA3-SpmCherry_n and pcDNA3-SpmCherry_full

The pcDNA3-SpmCherry_n plasmid was constructed as follows: the 5′-split mCherry (1–396) sequence was amplified using pcDNA3-EF1 α-mCherry as a template. The mouse β-actin intron III sequence was amplified by PCR using genomic DNA from C57/B6J mouse. The mCherry (367–426)-P2A peptide-coding sequence (5′-GGAAGCGGAGCTACTAACTTCAGCCTGCTGAAGCAGGCTGGAGACGTGGAGGAGAACCCTGGACCT-3′) was fused to the 5′-end of the puromycin resistance gene by PCR amplification. The C402T silent mutation for introducing the CRISPR-Cas12a PAM was added in the PCR primer. All fragments were fused and inserted into *Hind*III–*EcoR*I sites of pcDNA3 using NEBuilder HiFi DNA Assembly Master Mix (New England Biolabs), in accordance with the manufacturer’s protocol. For construction of the pcDNA3-SpmCherry_full plasmid, the 3′-split mCherry (427–708) sequence was amplified using pcDNA3-EF1α-mCherry as a template, and inserted into the *Hind*III site of pcDNA3-SpmCherry_n using NEBuilder HiFi DNA Assembly Master Mix (New England Biolabs), in accordance with the manufacturer’s protocol.

### pCriMGET_9-12a_SpmCherry_c

To construct the pCriMGET_9-12a_SpmCherry_c plasmid, the SpmCherry knock-in donor (402–708) that included 800-bp homology arms’ sequences was amplified by PCR using pcDNA3-SpmCherry_full as a template. Five silent mutations (T406A, C407G, C408T, C411T, C414T) were added to the PCR primer to introduce resistance against CRISPR-Cas9 and CRISPR-Cas12a cleavage. These fragments were fused and inserted into the *Sal*I and *BamH*I sites of the pCriMGET_9-12a multiple cloning site using NEBuilder HiFi DNA Assembly Master Mix, in accordance with the manufacturer’s protocol. Other donors, including those with homology arms of different lengths, were amplified by PCR using pCriMGET_9-12a_SpmCherry_c as a template. The fragments were fused and inserted into the *Sal*I and *BamH*I sites of the pCriMGET_9-12a multiple cloning site using NEBuilder HiFi DNA Assembly Master Mix.

### pCriMGET_9-12a_Hipp11-CAG-tdTomato-WPRE-pA

WPRE was amplified by PCR using the pCSII-EF-mRFP1-RfA plasmid (RIKEN BioResource Center) as a template, and inserted and fused into *EcoR*V and *Sac*I sites in pCAG-LSL-ZsGreen (Addgene #51269)^48^ using NEBuilder HiFi DNA Assembly Master Mix to exchange the ZsGreen and WPRE sequences. Then, oligoDNA (5′-TCGACCCGCCACCAATCATTTAAATAGGTCCCTCGACCTGCA-3′) was ligated into *Pst*I and *Sal*I sites in pCAG-LSL-WPRE-pA to eliminate the LSL sequence. The tdTomato coding sequence was amplified by PCR using tdTomato-C1 (Addgene #54653) as a template, and inserted and fused into *EcoR*I and *Kpn*I sites in pCAG-WPRE-pA using NEBuilder HiFi DNA Assembly Master Mix. The mouse genomic 500-bp upstream and downstream DNA sequences of the crRNA target site of the Hipp11 locus were amplified by PCR using the C57BL/6JJcl mouse genomic DNA as a template, and were used as left and right homology arms, respectively. The CAG-tdTomato-WPRE-pA fragment was purified from the *Spe*I-digested pCAG-tdTomato-WPRE-pA. Then, the three fragments were fused and inserted into *Kpn*I and *BamH*I sites of pCriMGET_9-12a using NEBuilder HiFi DNA Assembly Master Mix.

### pCriMGET_9-12a_Rosa26-CAG-LSL-NuM-mCherry-WPRE-pA

The NuM-mCherry (nuclear and membrane-mCherry: H2B-mCherry-P2A-mCherry-CAAX) reporter expression plasmid was constructed as follows: H2B and mCherry-P2A-mCherry-CAAX sequences were amplified by PCR using cDNA from HeLa cells and pT2A-DW-NuCyM (kindly gifted by Dr. Matsuda and Dr. Terai)^49^ as templates, respectively. The NuM sequence was purified from the *EcoR*V-digested pBluescript II SK(+)-NuM and ligated into the *EcoR*V site of pcDNA3. Then, the NuM sequence was amplified by PCR using pcDNA3-NuM as a template and inserted and fused into the *EcoR*V site of pCAG-LSL-WPRE-pA. The mouse genomic 500-bp upstream and downstream DNA sequences of the crRNA target site of the Rosa26 locus were amplified by PCR using C57BL/6JJcl mouse genomic DNA as a template, and were used as left and right homology arms, respectively. The two fragments were inserted and fused into *Xho*I and *Spe*I sites of pCriMGET_9-12a. Finally, the CAG-LSL-NuM-WPRE-pA sequence was purified from the *Spe*I-digested pCAG-LSL-NuM-WPRE-pA, and inserted and fused into *BamH*I and *EcoR*I sites of pCriMGET_9-12a using NEBuilder HiFi DNA Assembly Master Mix.

### pX330.1-syn-crRNA-TS-sgRNA, pX330.1-SpmCherry-sgRNA, pY094.1-syn-crRNA-TS-crRNA, and pY094.1-SpmCherry-crRNA

The pX330.1 plasmid was constructed as follows: The P2A-EGFP sequence was amplified by PCR from pCriMGET-3 × Flag-P2A-EGFP. Then, the fragment was inserted into the *EcoR*I site of pX330 (Addgene #42230)^7^ to fuse the Cas9 coding sequence using NEBuilder HiFi DNA Assembly Master Mix (New England Biolabs). The oligonucleotides syn-crRNA-TS-sgRNA (5′-GCTGTCCCCAGTGCATATTC-3′) and SpmCherry-sgRNA (5′-gTGCATTACGGGGCCGTCGGA-3′) were annealed and ligated into the *Bbs*I site of pX330.1. The pY094.1 plasmid was constructed as follows: the CBh promoter sequence was amplified by PCR from pX330. Then, the fragment was ligated into the *Mlu*I and *Kpn*I sites of pY094 (Addgene #84743)^50^ to exchange the cytomegalovirus (CMV) promoter. The oligonucleotides syn-crRNA-TS-sgRNA (5′-GCTGTCCCCAGTGCATATTCAGG-3′) and SpmCherry-sgRNA (5′-CCTCCGACGGCCCCGTAATGCAG-3′) were annealed and ligated into the *BsmB*I site of pY094.1.

### Cell culture and transfections

HEK293T cells (RIKEN BRC, RCB2202) were cultured in Dulbecco’s Modified Eagle’s Medium (Nissui) containing 10% fetal bovine serum (GIBCO), 4 mM L-glutamine (Nacalai Tesque), and 0.2% sodium bicarbonate. The HEK293T cells were transfected with plasmids using PEI MAX (Polysciences), in accordance with the manufacturer’s protocol.

### SpmCherry reconstitution system reporter cell line

HEK293T cells were transfected with *Sca*I-digested pcDNA3-SpmCherry_n and stably transduced cells were selected using puromycin (2 µg/mL). After 14 days of antibiotic selection, single colonies were isolated and PCR genotyped to confirm the insertion of the SpmCherry-Reporter sequence. Genomic DNA was extracted and purified using phenol–chloroform. SpmCherry-Reporter and AAVS1 locus genomic DNA sequences were amplified by PCR using the following primers: SpmCherry_F: 5′-tacgggccagatatacgcgttgac-3′ and SpmCherry_R: 5′-aggacagtgggagtggcaccttc-3′, and AAVS1_F: 5′-ttcttgtaggcctgcatcatcacc-3′ and AAVS1_R: 5′-atcctctctggctccatcgtaagc-3′. We established 22 reporter cell lines and used one cell line for the analysis.

### SpmCherry reconstitution assay

SpmCherry-Reporter HEK293T cells were transfected with pCriMGET_9-12a_SpmCherry_c, pX330.1-syn-crRNA-TS-sgRNA, and pX330.1-SpmCherry-sgRNA or pY094.1-syn-crRNA-TS-sgRNA and pY094.1-SpmCherry-sgRNA. At 72 h post-transfection, the cells were dissociated with PBS containing 0.25% trypsin and 0.53 mM EDTA (Nacalai Tesque) and mCherry expression was checked on a BD LSRFortessa X-20 flow cytometer. The data were analyzed using FlowJo Software (BD). For determination of SpmCherry reconstitution at the genomic level, single-cell clones of the mCherry-expressing cells were obtained by sorting mCherry-positive cells with FACS AriaIIIu cell sorter (BD), followed by colony picking. The genomic DNA was purified by phenol/chloroform extraction from each clone, and subjected to genotyping PCR with KOD One Blue PCR Master Mix (TOYOBO) using 10 ng of DNA as a template. The following primer pairs were used: SpmCherry_GT001: 5′-gcagagctctctggctaactagagaacc-3′, SpmCherry_GT002: 5′-ccttccagggtcaaggaaggcac-3′.

### crRNA, tracrRNA, gRNA, and Cas9 and Cas12a proteins

For the CRISPR-Cas9 system, we used syn-crRNA-TS-crRNA (5′-GCUGUCCCCAGUGCAUAUUCguuuuagagcuaugcu-3′), Hipp11-crRNA (5′-GAACACUAGUGCACUUAUCCguuuuagagcuaugcu-3′), Rosa26-crRNA (5′-ACUCCAGUCUUUCUAGAAGAguuuuagagcuaugcu-3′), Alt-R CRISPR-Cas9 tracrRNA (Integrated DNA Technologies, IDT#1072532), and Sp HiFi Cas9 nuclease V3 (Integrated DNA Technologies, IDT#1081060) in this study. The crRNA and tracrRNA were annealed (gRNAs) in a thermocycler (95 °C for 5 min, ramped down to 77 °C at 2 °C/s, then ramped down to 25 °C at 0.1 °C/s) and then stocked as 50 µM gRNAs. Upper- and lower-case letters indicate sequences of the target-specific protospacer region and the tracrRNA fusion domain, respectively. For the CRISPR-Cas12a system, we prepared syn-crRNA-TS-crRNA (5′-uaauuucuacucuuguagauGCUGUCCCCAGUGCAUAUUCAGG-3′), Rosa26-crRNA (5′-uaauuucuacucuuguagauUAGAAGAUGGGCGGGAGUCUUCU-3′), and Alt-R SpCas12a nuclease V3 (Integrated DNA Technologies, IDT#1076158). Here, upper- and lower-case letters indicate sequences of the target-specific protospacer region and the loop domain, respectively. The Cas9-crRNA, tracrRNA, Cas12a-crRNA, and Cas nucleases were obtained from Integrated DNA Technologies.

### Pronuclear injection

Pronuclear injection was performed as described previously^32^ with modifications. Briefly, gRNA and Cas nuclease were mixed and incubated for 15 min at room temperature to form a ribonucleoprotein complex. Then, pCriMGET_9-12a_donor was added and the mixture was centrifuged at 20,400*g* for 30 min at 4 °C. The supernatant was microinjected into the pronuclei of zygotes. To generate *Hipp11*^*CAG-tdTomato*^ knock-in mice, a cocktail containing pCriMGET_9-12a-Hipp11-CAG-tdTomato-WPRE-pA (25 ng/µL), Hipp11-gRNA (2 µM), syn-crRNA-TS-gRNA (1 µM), and Cas9 protein (50 ng/µL) was microinjected into the pronuclei of zygotes. To generate *Rosa26*^*CAG-LSL-NuM*^ knock-in mice, a cocktail for CRISPR-Cas12a containing pCriMGET_9-12a-Rosa26-CAG-LSL-NuM-WPRE-pA (25 ng/µL), Rosa26-gRNA (2 µM), syn-crRNA-TS-gRNA (1 µM), and Cas12a protein (50 ng/µL) or a cocktail for CRISPR-Cas9 containing pCriMGET_9-12a-Rosa26-CAG-LSL-NuM-WPRE-pA (25 ng/µL), Rosa26-gRNA (2 µM), syn-crRNA-TS-gRNA (1 µM), and Cas9 protein (50 ng/µL) was microinjected into the pronuclei of zygotes.

### Animals used in this study

C57BL/6JJmsSLC and Jcl:ICR mice were obtained from Japan SLC Inc. (Shizuoka, Japan) and CLEA Japan Inc. (Tokyo, Japan), respectively. The *Tbx3*^*CreERT2*^ strain was generated previously^35^. All of the experiments were performed in accordance with ARRIVE guidelines (https://arriveguidelines.org) and the guidelines of the Kyoto University Regulation on Animal Experimentation, and were approved by the Committee for Animal Experiments of the Institute for Life and Medical Sciences, Kyoto University (A21-2-2).

### Genotyping PCR for blastocysts and mouse tail

Blastocysts and mouse tail samples were prepared for genotyping PCR as described previously^32^. The PCR was carried out with 1–2 µL lysis samples using KOD One Blue PCR Master Mix (TOYOBO). To genotype the *Hipp11*^*CAG-tdTomato*^ knock-in and *Rosa26*^*CAG-LSL-NuM*^ knock-in blastocysts and mouse tail samples, the following primer pairs were used: 3′ junction of *Hipp11*^*CAG-tdTomato*^ (Hipp11-CAG-tdTomato KI_GT001: 5′-gccatcatgctctcactgcctc-3′, Hipp11-CAG-tdTomato KI_GT002: 5′-gaatctctggctggccttgctc-3′, and Hipp11-CAG-tdTomato KI_GT003: 5′-ccctcagacgagtcggatctcc-3′), 5′ junction of *Hipp11*^*CAG-tdTomato*^ (Hipp11-CAG-tdTomato KI_GT004: 5′-tgaaagtagcttgtggcaagtatcaagg-3′, Hipp11-CAG-tdTomato KI_GT005: 5′-cctgttccatcagcttcagcctg-3′, and CAG_Rev: 5′-tcatgtactgggcataatgccagg-3′), 3′ junction of *Rosa26*^*CAG-LSL-NuM*^ (Rosa26-CAG-LSL-NuM_GT001: 5′-ctgcctcctggcttctgaggac-3′, Rosa26-CAG-LSL-NuM_GT002: 5′-ttgaggccccagctacagcctc-3′, and Rosa26-CAG-LSL-NuM_GT003: 5′-ccctcagacgagtcggatctcc-3′), and 5′ junction of *Rosa26*^*CAG-LSL-NuM*^ (Rosa26-CAG-LSL-NuM_GT004: 5′-ctcgtcgtctgattggctctcg-3′, Rosa26-CAG-LSL-NuM_GT005: 5′-ttcaattcccctgcaggacaacg-3′, and CAG_Rev). To genotype the Tbx3^creERT2^ knock-in mice, the following primer pairs were used: Tbx3creERT2_GT001: 5′-cctctggctcagtgtccttgtcac-3′, Tbx3creERT2_GT002: 5′-tggcagggcctgtggtatctagc-3′, and Tbx3creERT2_GT003: 5′-acgtatatcctggcagcgatcgc-3′.

### Observation of tdTomato fluorescence

The expression of tdTomato in F_0_ 4-week-old mouse was examined by the naked eye using Handy Green Pro Plus (540) LED light and red goggles (RelyOn Ltd.). After anesthetization, photos were taken of the whole body and tissues under 540-nm excitation light from a RelyOnTwin-LED Light using a Canon EOS Kiss X10 digital single-lens reflex camera (Canon Co. Ltd.) with a RelyOn 600-nm LP filter.

### Real-time PCR analysis

For determination of the *tdTomato* gene expression in F_0_ 4-week-old *Hipp11*^*CAG-tdTomato*^ knock-in mouse, real-time PCR was performed on RNA from the brain, heart, lung, liver, kidney, spleen, intestine, and skeletal muscle tissue. RNA from each tissue was extracted using ISOGEN (NIPPON GENE), in accordance with the manufacturer’s protocol. Samples were treated with DNase I (Takara) at 37 °C for 20 min, after which RNAs were purified with RNeasy Mini kit (Qiagen) for clean-up, as per the manufacturer’s protocol. cDNAs were synthesized by using ReverTra Ace^®^ qPCR RT Master Mix (TOYOBO) and applied to a qPCR reaction mixture (20 µL) of the THUNDERBIRD SYBR qPCR Mix (TOYOBO). The reactions were performed in duplicate for each sample using the Applied Biosystems 7500 Real-Time system (Applied Biosystems). The following primer pairs were used: tdTomato_qPCR_Fw: 5′-atcgtggaacagtacgagcg-3′, tdTomato_qPCR_Rev: 5′-tgaactctttgatgacggcca-3′, and G3pdh_qPCR_Fw: 5′-aggtcggtgtgaacggatttg-3′, and G3pdh_qPCR_Rev: 5′-tgtagaccatgtagttgaggtca-3′.

### Immunohistochemistry

Livers and plantar skin tissues from 6-week-old *Rosa26*^*CAG-LSL-NuM-mCherry*^;*Tbx3*^*CreERT2*^ mice that had been administered tamoxifen or corn oil were collected and cryoprotected in PBS containing 20% sucrose and frozen in optimal cutting temperature compound. The samples were sectioned, immunostained, fixed with 4% paraformaldehyde, and permeabilized with 0.5% Triton X-100 in Tris-buffered saline for 15 min at room temperature. Then, the sections were blocked with Blocking-One Histo (Nacalai Tesque) at room temperature for 1 h, incubated with primary antibodies at 4 °C overnight, washed, and incubated for 1 h with secondary antibodies. The samples were mounted using VECTASHIELD PLUS Antifade Mounting Medium with DAPI (Vector Laboratories). The primary antibodies were anti-Tbx3 (rabbit, 1:200, ab99302; Abcam), anti-mCherry (chicken, 1:1000, ab205402; Abcam), anti-HNF4a (goat, 1:500, sc-6556; Santa Cruz Biotechnology), and anti-CD49f (rat, 1:500, 313602; Biolegend). The secondary antibodies were Alexa Fluor 488-conjugated anti-rabbit, Cy3-conjugated anti-chicken, Alexa647-conjugated anti-mouse, and Alexa647-conjugated anti-rat (Jackson ImmunoResearch, West Grove, PA, USA). All images were acquired using an Olympus FV3000 confocal microscope.

### In vitro Cas9- or Cas12a-nuclease cleavage assay

The DNA cleavage activity was assayed using pCriMGET_9-12a_SpmCherry_c plasmid as a substrate. Mixtures of ribonucleoprotein complex (syn-crRNA-TS-crRNA:tracrRNA (Integrated DNA Technologies, IDT#1072532) and Sp HiFi Cas9 nuclease V3 (Integrated DNA Technologies, IDT#1081060), or syn-crRNA-TS-crRNA and Alt-R SpCas12a nuclease V3 (Integrated DNA Technologies, IDT#1076158) were added into reaction tubes together with 10 × NEB buffer 3.1 and pCriMGET_9-12a_SpmCherry_c plasmid. The mixtures were incubated at 37 °C for 1 h, followed by treatment with 20 mg/mL Proteinase K (Nacalai Tesque) for 10 min at 56 °C to release the DNA substrate from the Cas nuclease. The products of each reaction were electrophoresed on 1.0% agarose gel. As a single digestion control, EcoRV-HF (NEB) digested plasmid DNA was used.

## Supplementary Information

Supplementary Information.

## Data Availability

All data generated or analyzed during this study are included in this published article and available from the corresponding author on reasonable request.
