# Supplementary material for: Development of an in vivo cleavable donor plasmid for targeted transgene integration by CRISPR-Cas9 and CRISPR-Cas12a
Source: Sci Rep. 2022 Oct 22;12:17775. doi: 10.1038/s41598-022-22639-6 (PMC9588054; doi:10.1038/s41598-022-22639-6)
Supplement: Supplementary file 1 — Supplementary Information. [file 41598_2022_22639_MOESM1_ESM.pdf]

Supplementary information

Development of an *in vivo* cleavable donor plasmid for targeted transgene integration by CRISPR-Cas9 and CRISPR-Cas12a

Riki Ishibashi[1,2]*, Ritsuko Maki[1], Satsuki Kitano[3],
Hitoshi Miyachi[3] and Fumiko Toyoshima[1,2]*

[1]Department of Biosystems Science, Institute for Life and Medical Sciences, Kyoto University, Sakyo-ku, Kyoto 606-8507, Japan

[2]Department of Mammalian Regulatory Networks, Graduate School of Biostudies, Kyoto University, Sakyo-ku, Kyoto 606-8502, Japan

[3]Reproductive Engineering Team, Institute for Life and Medical Sciences, Kyoto University, Sakyo-ku, Kyoto 606-8507, Japan

*Corresponding authors:
Riki Ishibashi
E-mail: rishibas@infront.kyoto-u.ac.jp
Tel.: +81-75-751-4016
Fax: +81-75-751-4037

Fumiko Toyoshima
E-mail: ftoyoshi@infront.kyoto-u.ac.jp
Tel.: +81-75-751-4015
Fax: +81-75-751-4037

This PDF file includes :
Supplementary Figure S1 to S10
Supplementary Table S1

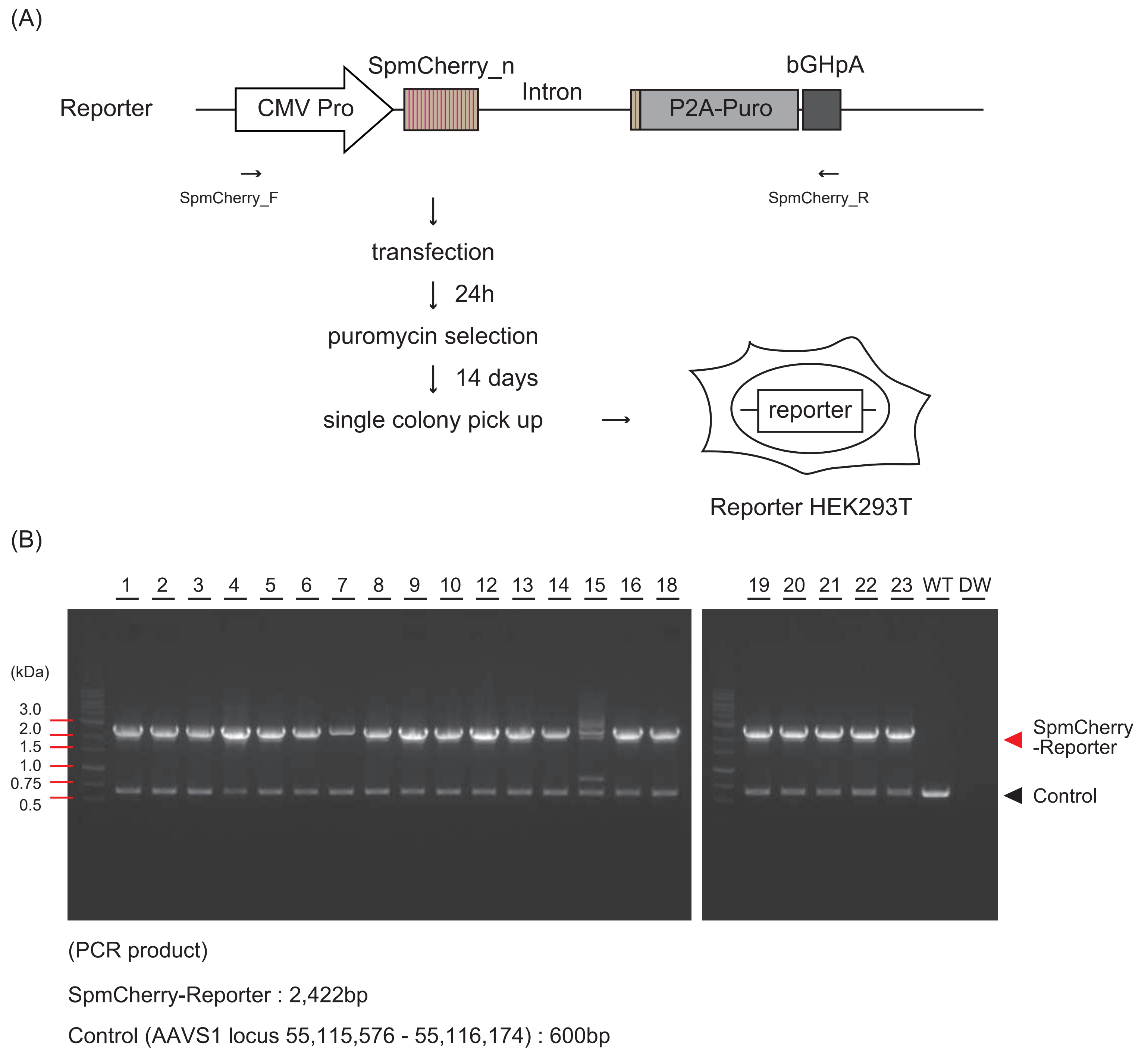

Supplementary Figure S1. Generation of Split-mCherry (SpmCherry) reconstitution reporter cells. (A) Design of CMV-SpmCherry-P2A-Puro-pA reporter sequence. The mCherry coding sequence (1–300) was divided by the mouse ActB intron III sequence. (B) Genotyping PCR in 23 SpmCherry reporter HEK293T cell lines. The gel electrophoresis images show the PCR products of the SpmCherry reporter (2,422 bp) using the SpmCherry_F/R primers shown in (A) and an internal control (AAVS1 locus; 55,115,576–55,116,174: 599 bp).

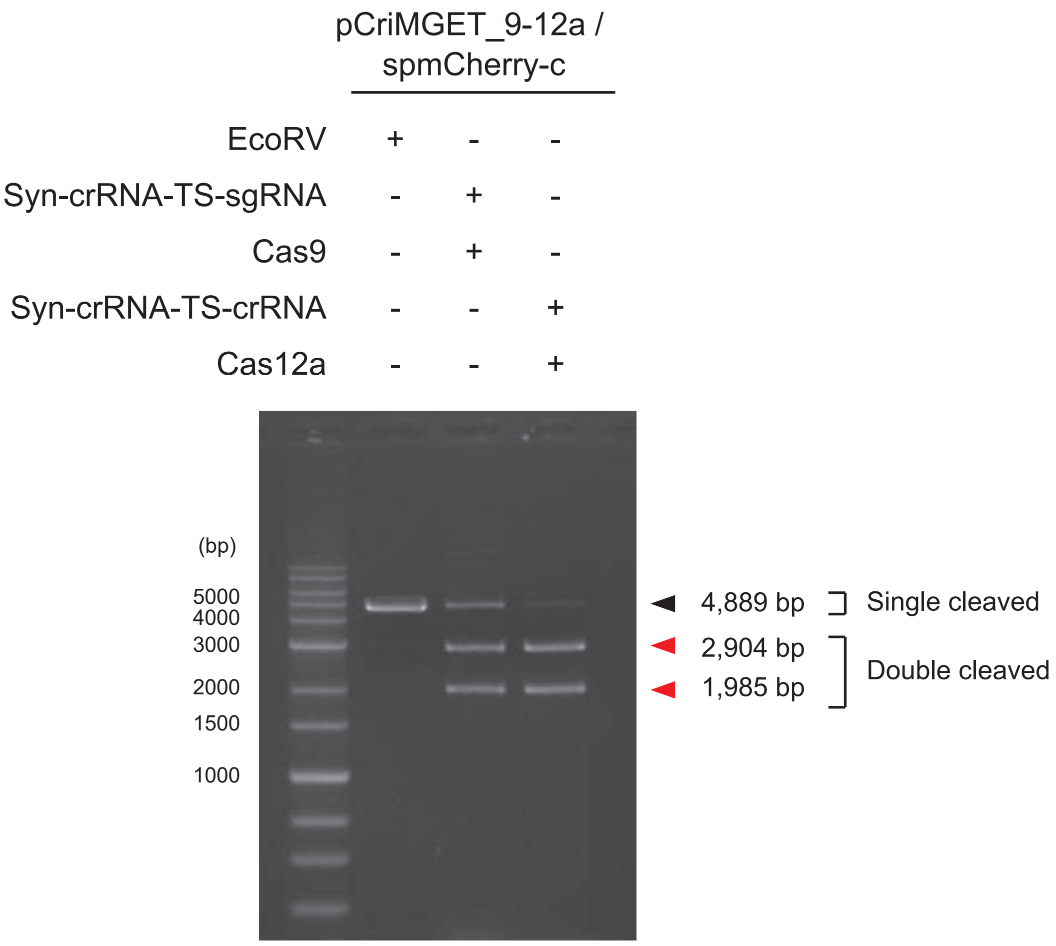

Suplementary Figure S2. ***In vitro*** **pCriMGET_9-12a cleavage using Cas9 or Cas12a nuclease.** pCriMGET_9-12a_SpmCherry_c was incubated with ribonuclease complex of syn-crRNA-TS-sgRNA and Cas9 nuclease, or syn-crRNA-TS-crRNA and Cas12a nuclease. Samples were separated by 1.0% agarose gel electrophoresis. Black arrowhead shows a single cleavage band and red arrowhead shows double cleavage bands. EcoRV digested product was used as a single cleavage marker.

(A)

(B)

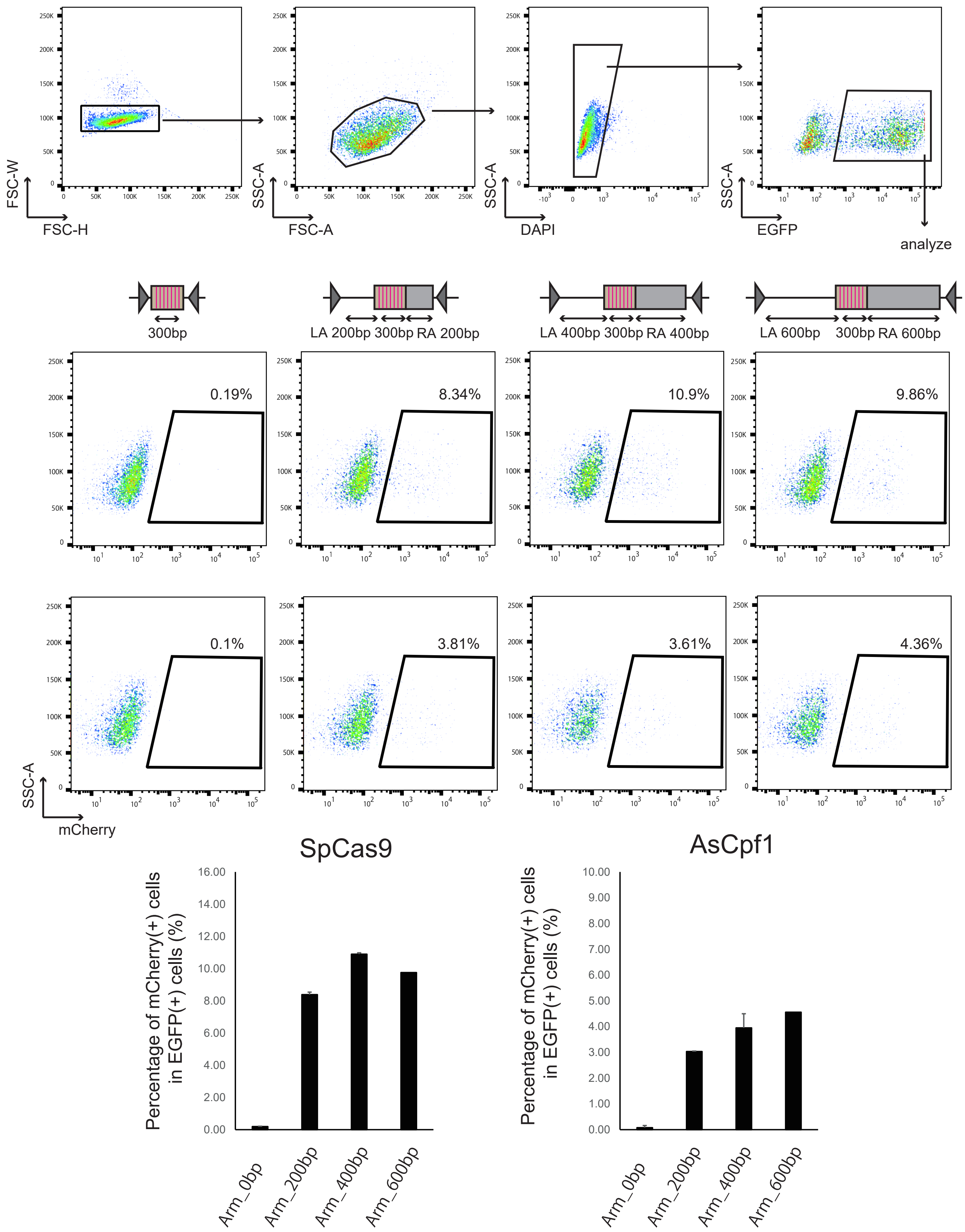

Supplementary Figure S3. Effect of the length of the homology arms on knock-in frequency.

(A) Gating strategy for fluorescence-activated cell sorting (FACS) analysis. Single cells were gated by FSC-H vs FSC-W. Live cells were gated by the DAPI- population. $EGFP^{high}$ cells were gated and the percentages of $mCherry^{+}$ cells were calculated in the $EGFP^{high}$ cell population. (B) FACS analyses of mCherry expression in the Split-mCherry reporter cells transfected with the indicated length of donor sequence. (upper, pCriMGET_9-12a/CRISPR-Cas9 system; lower, pCriMGET_9-12a/CRISPR-Cas12a system). Average percentages of $mCherry^{+}$ cells in each sample are shown in the bar graph (mean ± s.d. from three experiments).

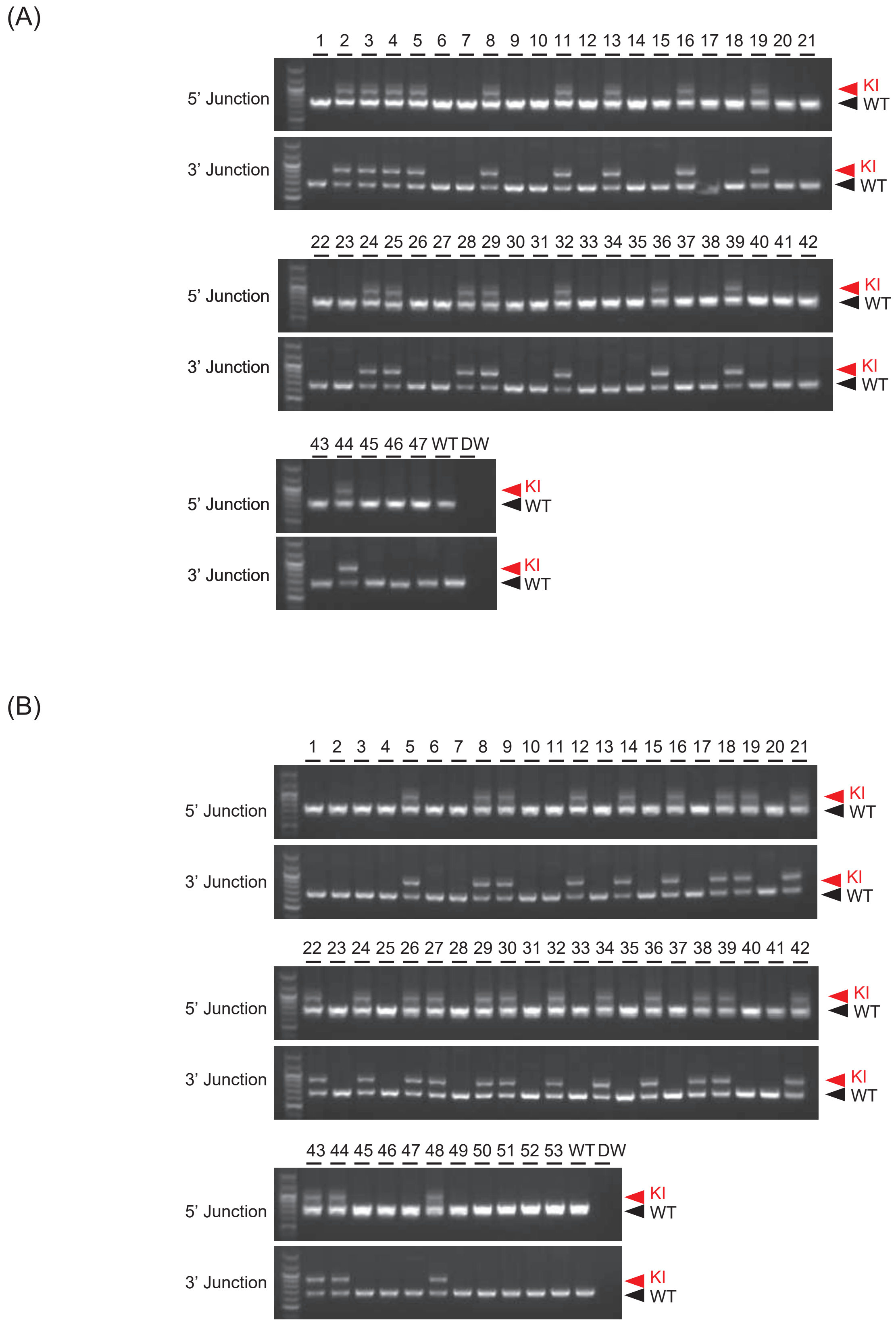

Supplementary Figure S4. Generation of *Hipp11*$^{CAG\text{-}tdTomato}$ knock-in mice by the pCriMGET and pCriMGET_9-12a systems. Genotyping PCR analyses of *Hipp11*$^{CAG\text{-}tdTomato}$ knock-in blastocysts generated by pCriMGET (A) and pCriMGET_9-12a (B). KI and WT bands for 5′ and 3′ junctions are indi -cated by red and black arrowheads, respectively.

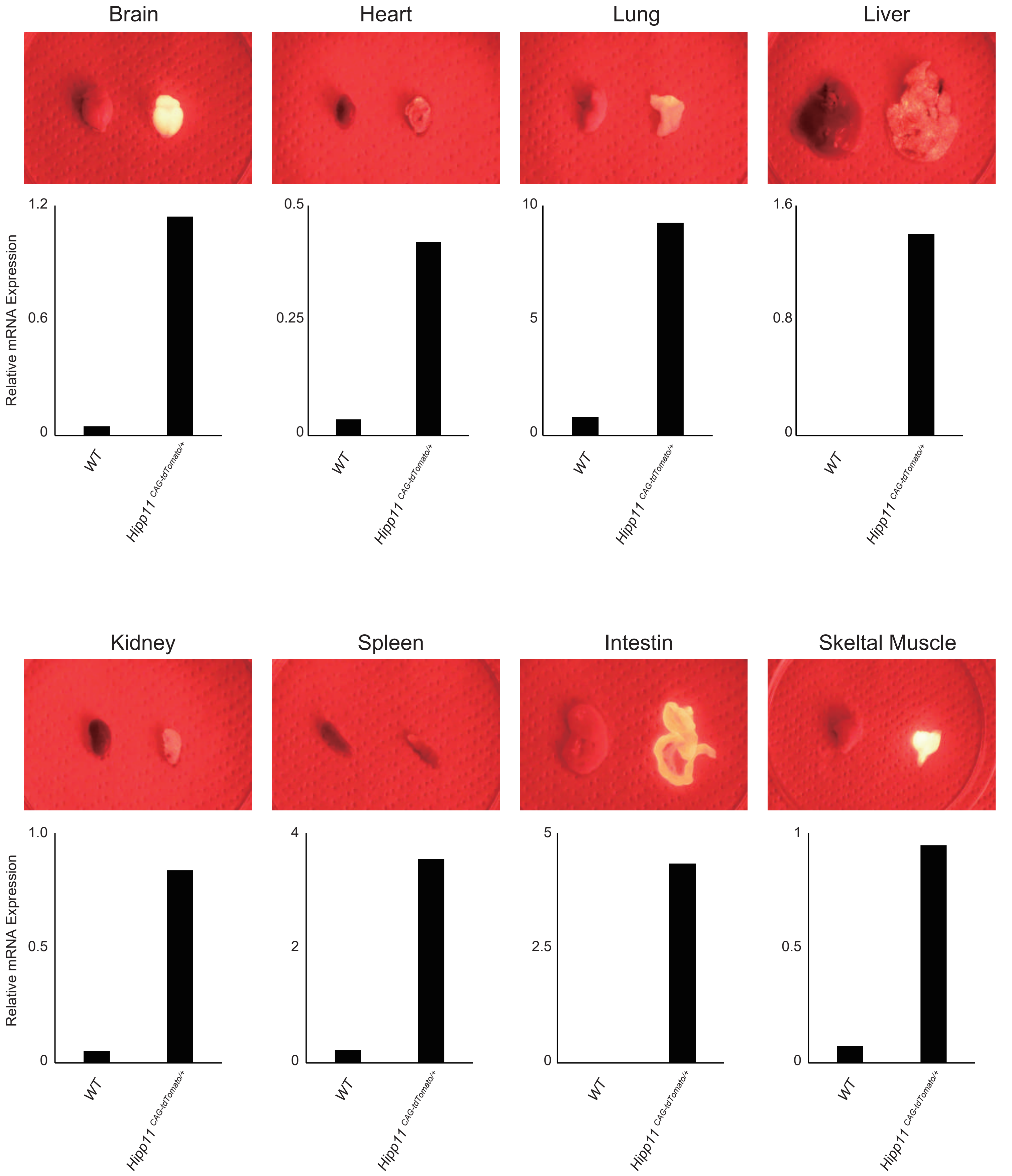

Supplementary Figure S5. tdTomato expression in tissues of *Hipp11*$^{CAG\text{-}tdTomato}$ *KI*/+ mice.

Images of tdTomato expression in brain, heart, lung, liver, kidney, spleen, intestine, and skeletal muscle. Tissues on the left and right are from WT and *Hipp11*$^{CAG\text{-}tdTomato}$ *KI*/+ mice, respectively. Images were taken under 540-nm excitation light using a single-lens reflex camera with a 600-nm LP filter. qPCR analyses for expression of *tdTomato* mRNA are shown at the bottom. For qPCR, values were normalized to expression of *G3pdh*.

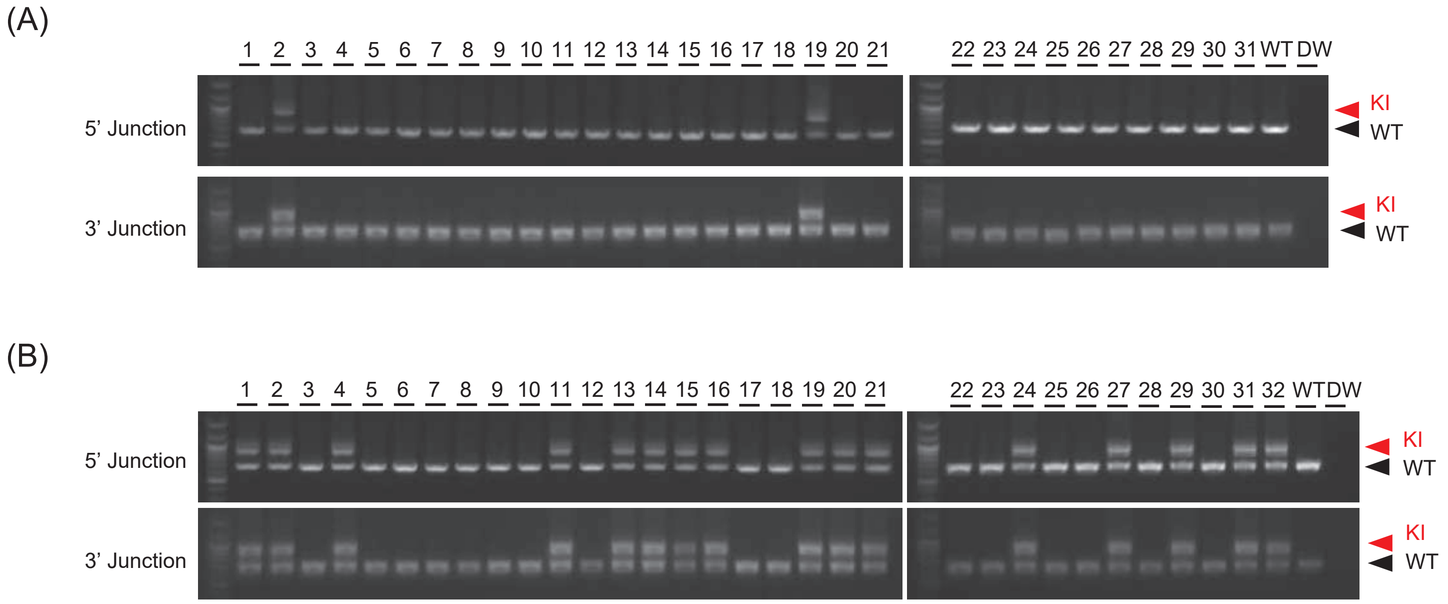

Sample (Cas9)	KI frequency (%)
Syn-crRNA-TS-crRNA(-)	6.5% (2/31 Blastocyst)
Syn-crRNA-TS-crRNA(+)	50% (16/32 Blastocyst)

Supplementary Figure S6. Generation of *Rosa26$^{CAG\text{-}LSL\text{-}NuM\text{-}mCherry}$* knock-in mice by pCriMGET_9-12a and CRISPR-Cas9 with or without syn-crRNA-TS-crRNA. Genotyping PCR analyses of *Rosa26$^{CAG\text{-}LSL\text{-}NuM\text{-}mCherry}$* knock-in blastocysts generated by pCriMGET_9-12a and CRISPR-Cas9 in the absence (A) or presence (B) of syn-crRNA-TS-crRNA. KI and WT bands for 5′ and 3′ junctions are indicated by red and black arrowheads, respectively. The table shows the knock-in frequency in the blastocysts.

(A)

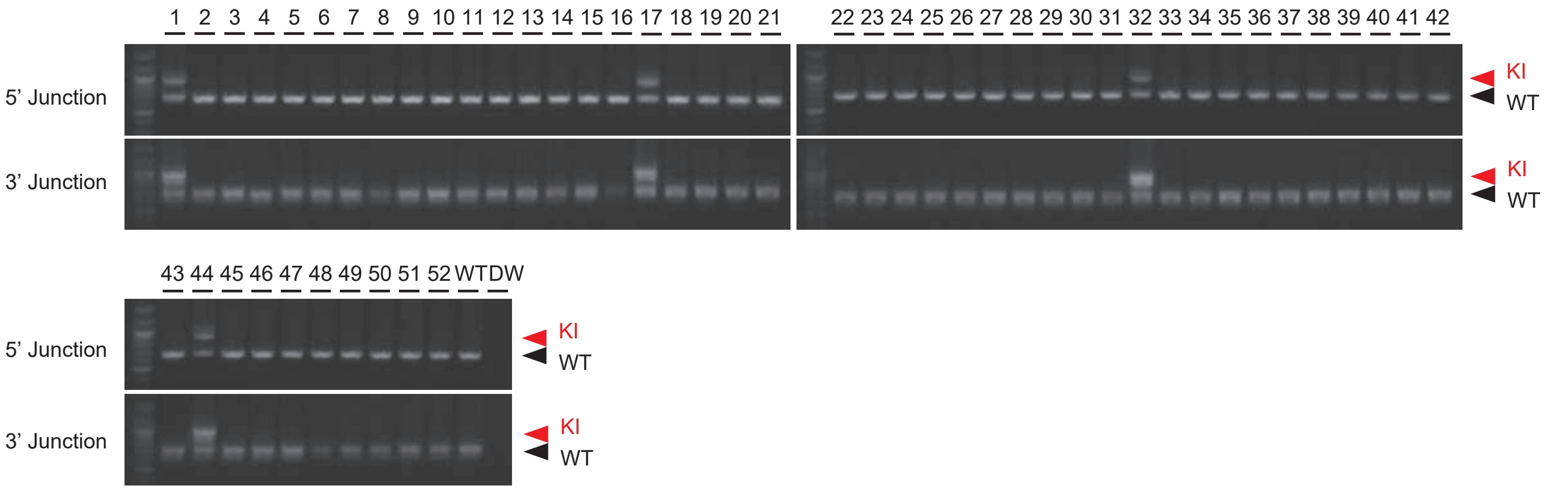

(B)

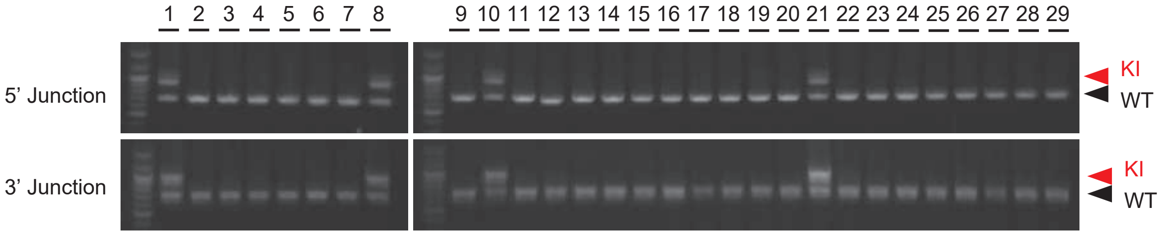

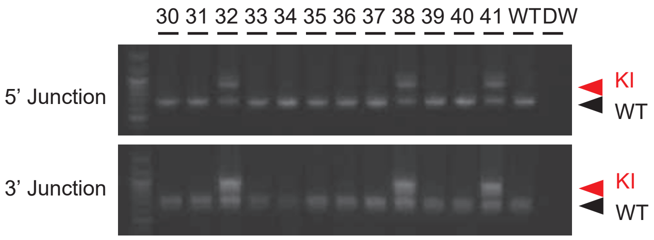

Sample (Cas12a)	KI frequency (%)
Syn-crRNA-TS-crRNA(-)	7.7% (4/52 Blastocyst)
Syn-crRNA-TS-crRNA(+)	17% (7/41 Blastocyst)

Supplementary Figure S7. Generation of *Rosa26*$^{CAG\text{-}LSL\text{-}NuM\text{-}mCherry}$ knock-in mice by pCriMGET_9-12a and CRISPR-Cas12a with or without syn-crRNA-TS-crRNA. Genotyping PCR analysis of *Rosa26*$^{CAG\text{-}LSL\text{-}NuM\text{-}mCherry}$ knock-in blastocysts generated by pCriMGET_9-12a and CRISPR-Cas12a in the absence (A) or presence (B) of syn-crRNA-TS-crRNA. KI and WT bands for 5′ and 3′ junctions are indicated by red and black arrowheads, respectively. The table shows the knock-in frequency in the blastocysts.

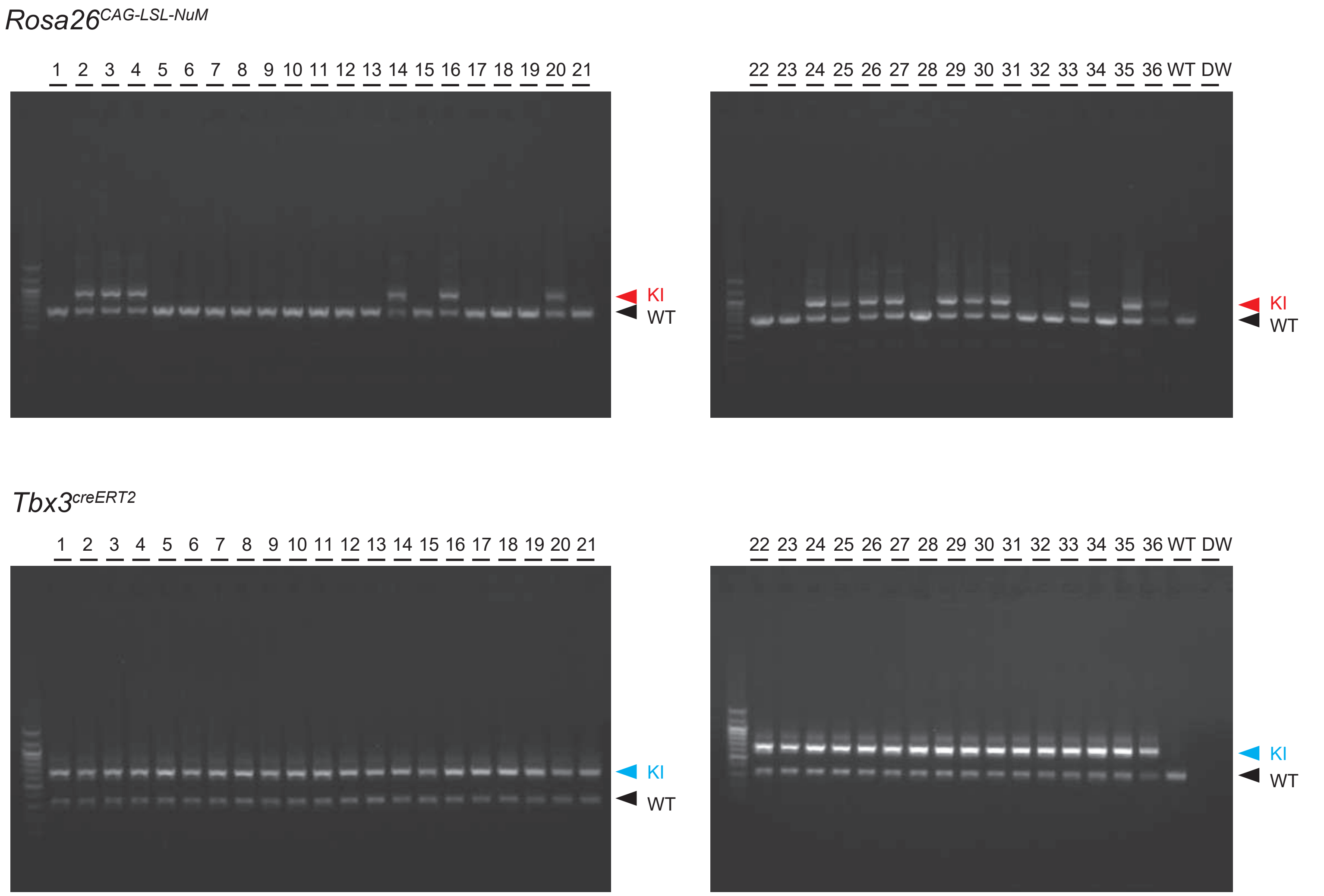

Supplementary Figure S8. Germline transmission of the donor gene in *Rosa26*$^{CAG\text{-}LSL\text{-}NuM\text{-}mCherry}$ knock-in mice. Genotyping PCR of 4-week-old F$_1$ pups derived from a *Rosa26*$^{CAG\text{-}LSL\text{-}NuM\text{-}mCherry}$ hemizygous knock-in male mouse and *Tbx3*^{CreERT2} homozygous knock-in female mice. Upper panel shows *Rosa26*$^{CAG\text{-}LSL\text{-}NuM\text{-}mCherry}$ knock-in (KI) (934 bp) and wild-type (WT) (677 bp) bands; lower panel shows *Tbx3*^{CreERT2} KI (657 bp) and WT (335 bp) bands.

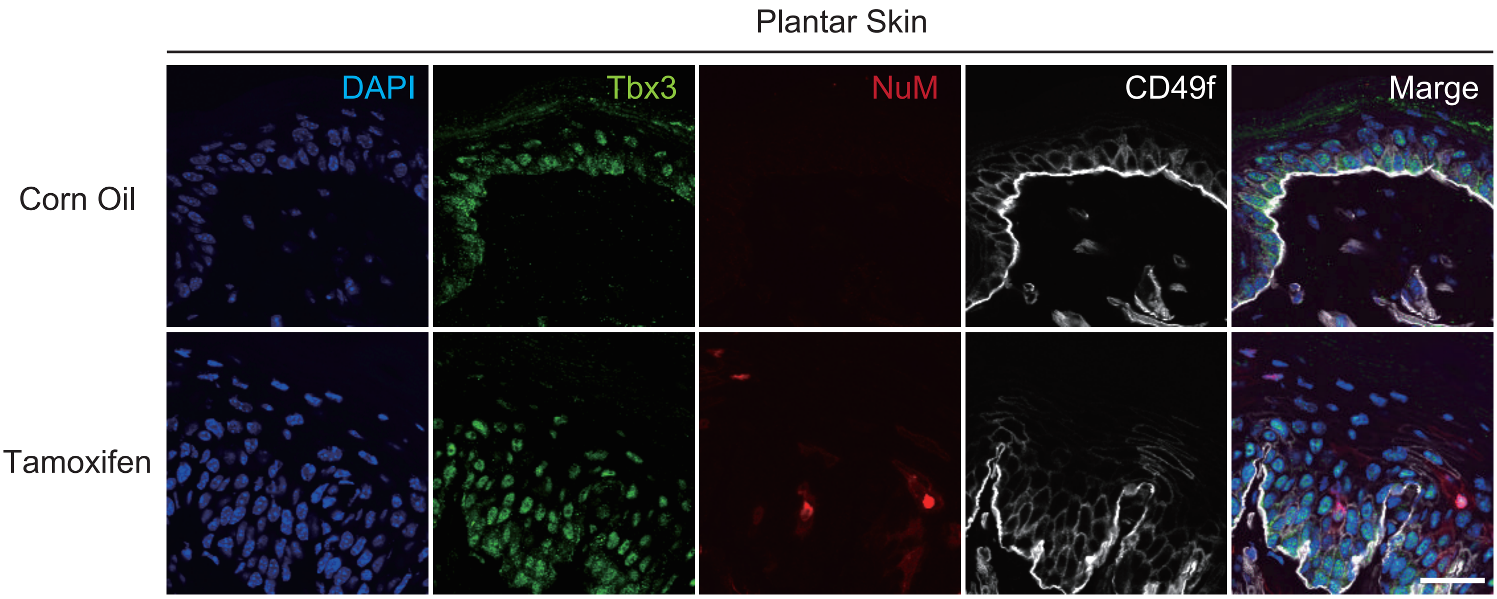

Supplementary Figure S9. Donor transgene expression in plantar skin of *Rosa26*$^{CAG\text{-}LSL\text{-}NuM\text{-}mCherry}$ knock -in mice. Representative immunofluorescence images of plantar skin tissue in the *Rosa26*$^{CAG\text{-}LSL\text{-}NuM\text{-}mCherry}$; *Tbx3*^{CreERT2} mice administered control corn oil and tamoxifen. Tbx3 (green), NuM-mCherry (red), CD49f (white), and DAPI (blue). Scale bar, 20μm.

Supplementary Table S1. Mismatches of the syn-crRNA-TS-crRNA_9-12a sequence for CRISPR-Cas9 and -Cas12a in the mouse and human genomes.

	Reference genome	mismatches			
		0	1	2	3
Cas9	mouse genome (GCRm38/mm10)	0	0	0	5
	human genome (GCRh38/hg 38)	0	0	0	0
Cas12a	mouse genome (GCRm38/mm10)	0	0	0	0
	human genome (GCRh38/hg 38)	0	0	0	0

The number of syn-crRNA-TS_9-12a mismatches (0–3 nucleotides) for CRISPR-Cas9 and -Cas12a, and the mismatched bases are shown.

Related to figure 2

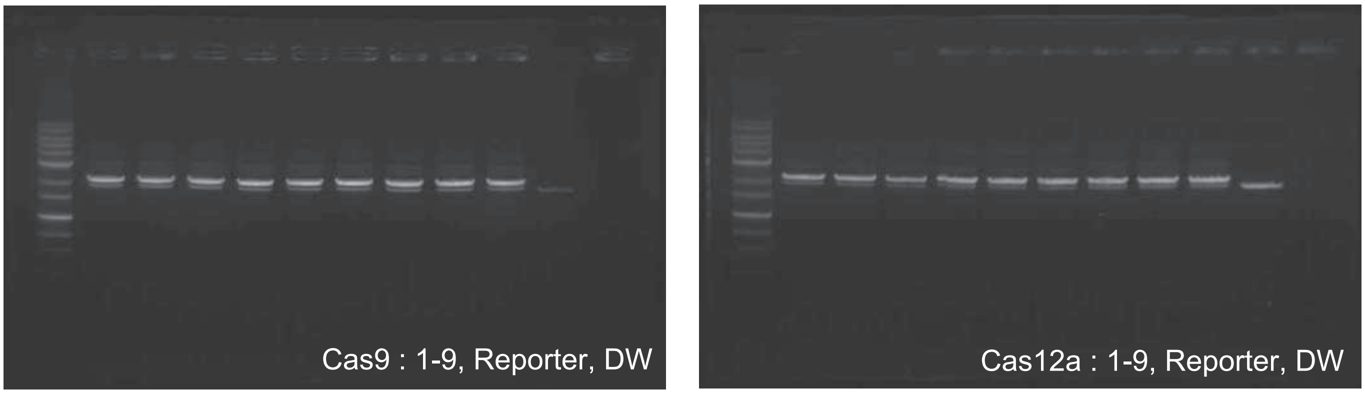

Related to figure 3

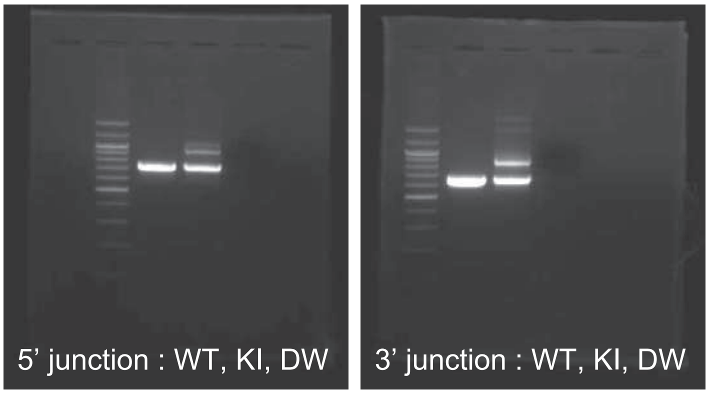

Related to figure 4

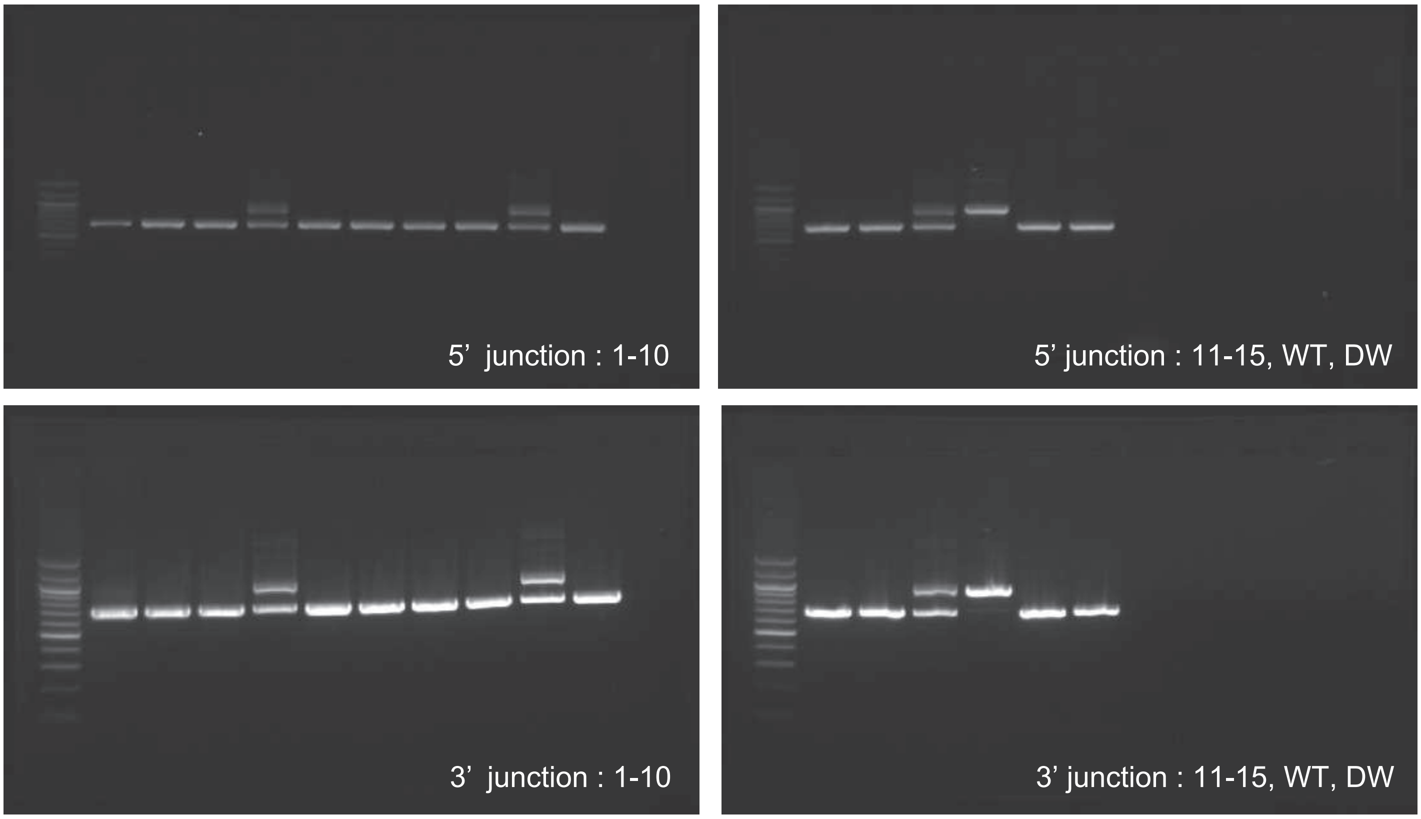

Related to supplementary figure S1

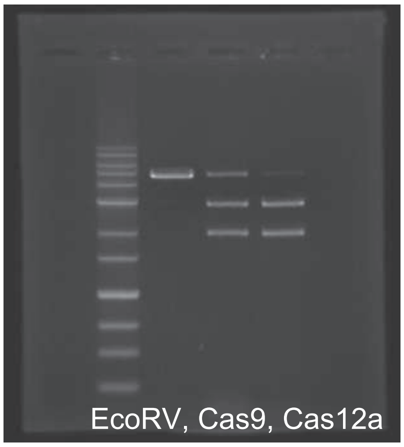

Related to supplementary figure S2

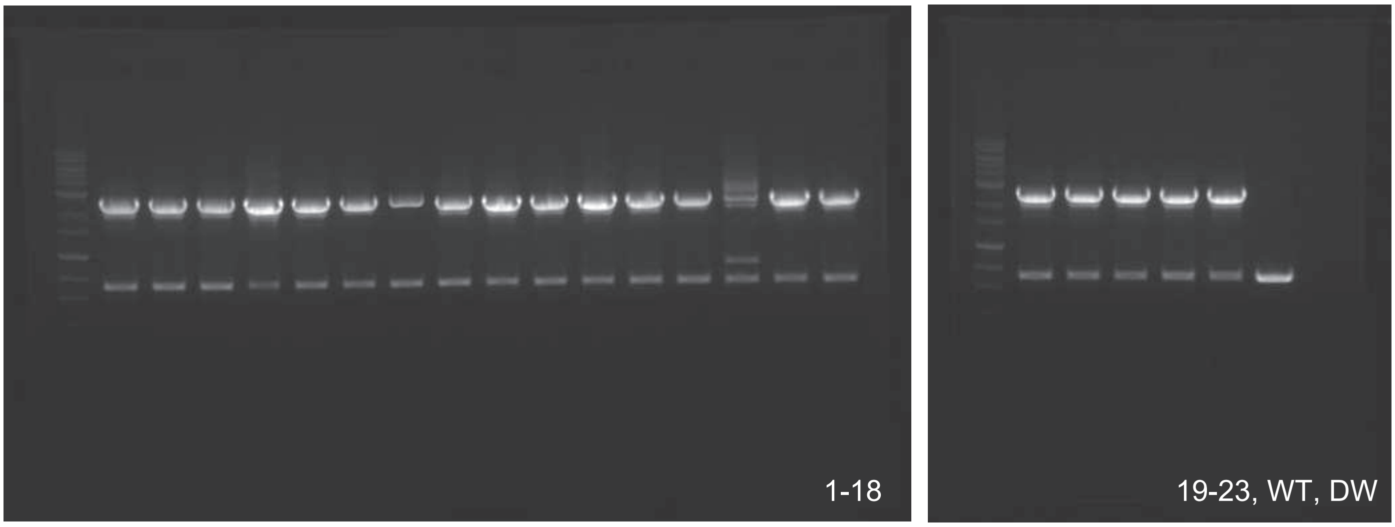

Related to supplementary figure S4 (A)

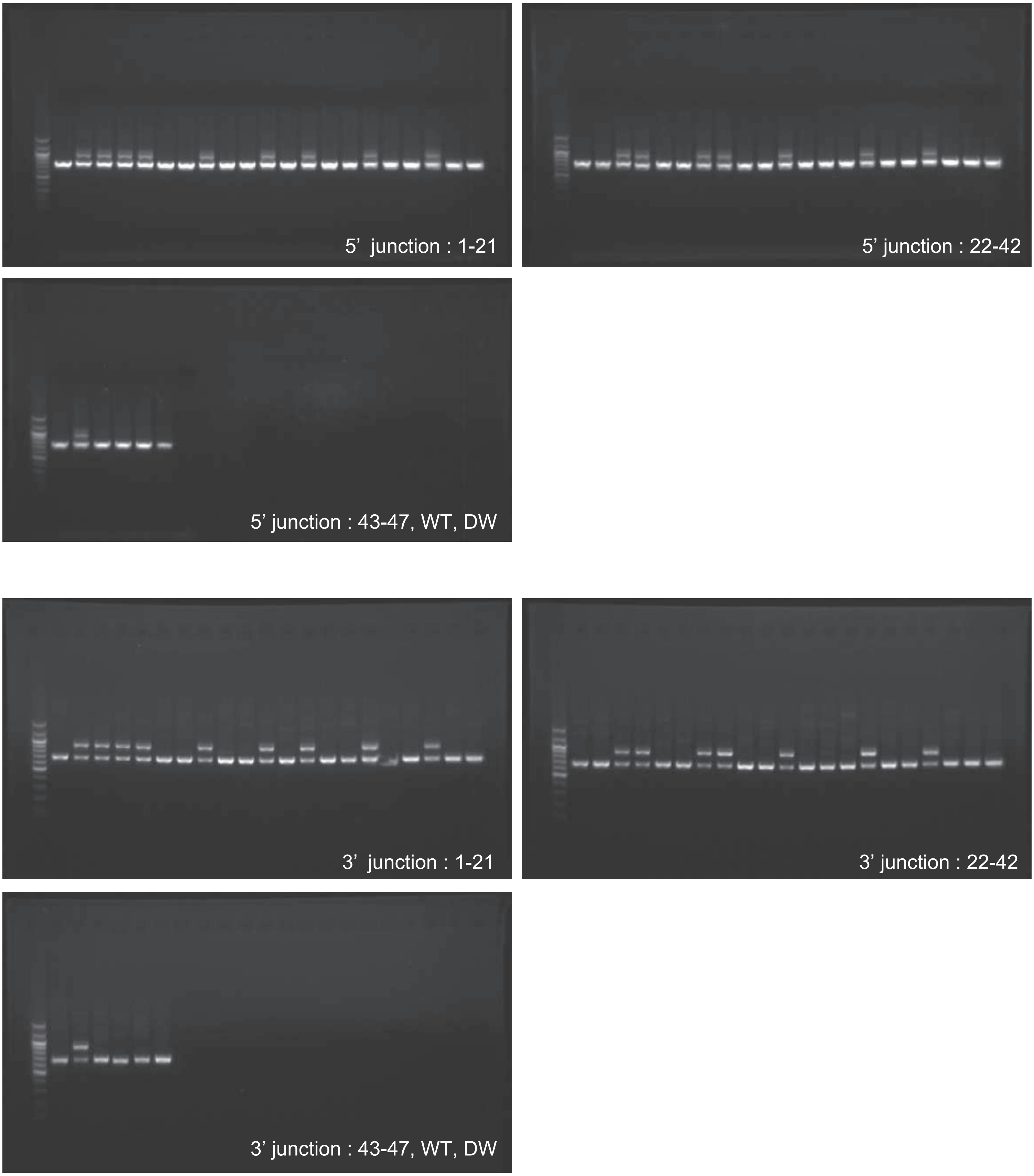

Related to supplementary figure S3 (B)

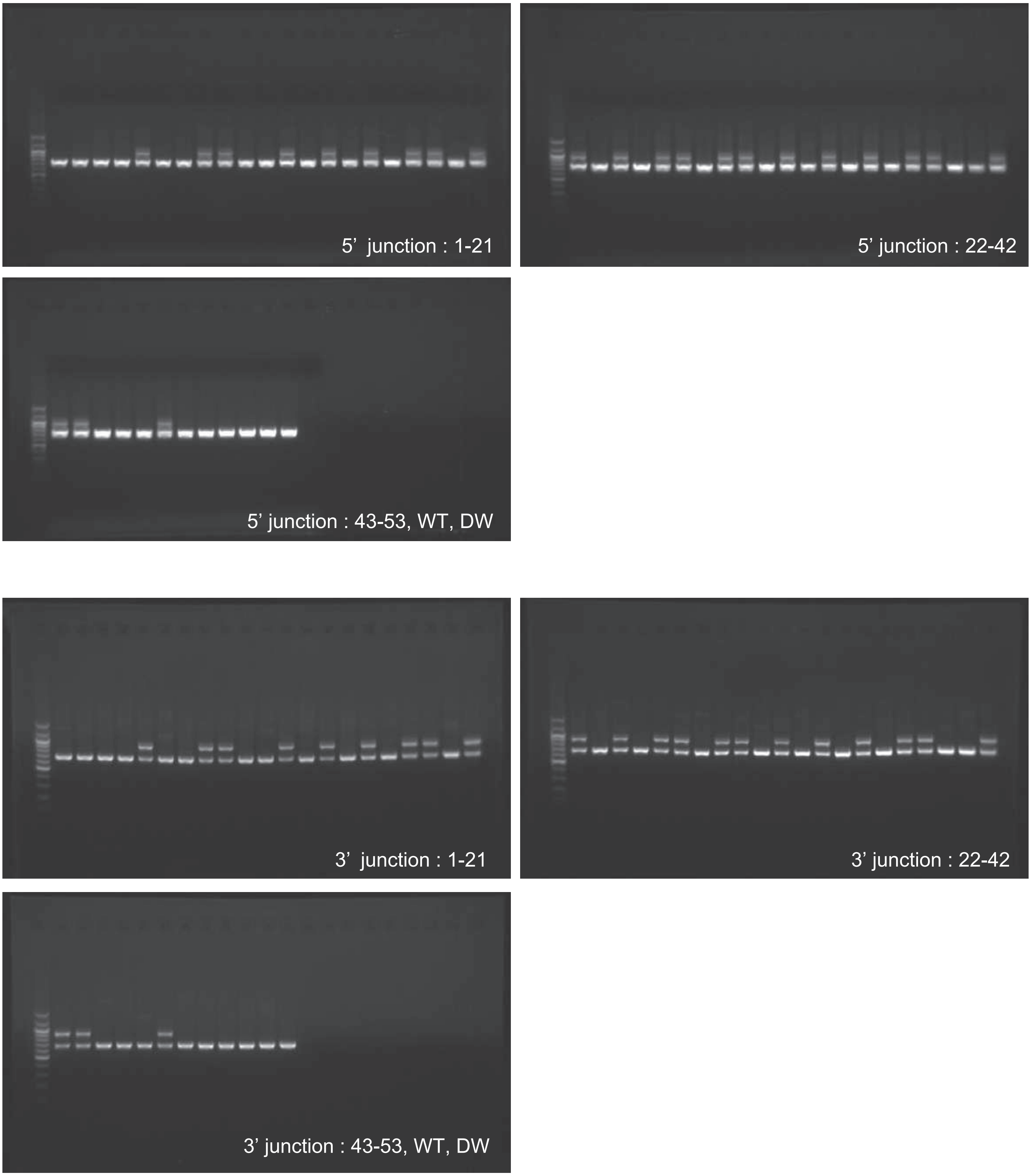

Related to supplementary figure S4 (A)

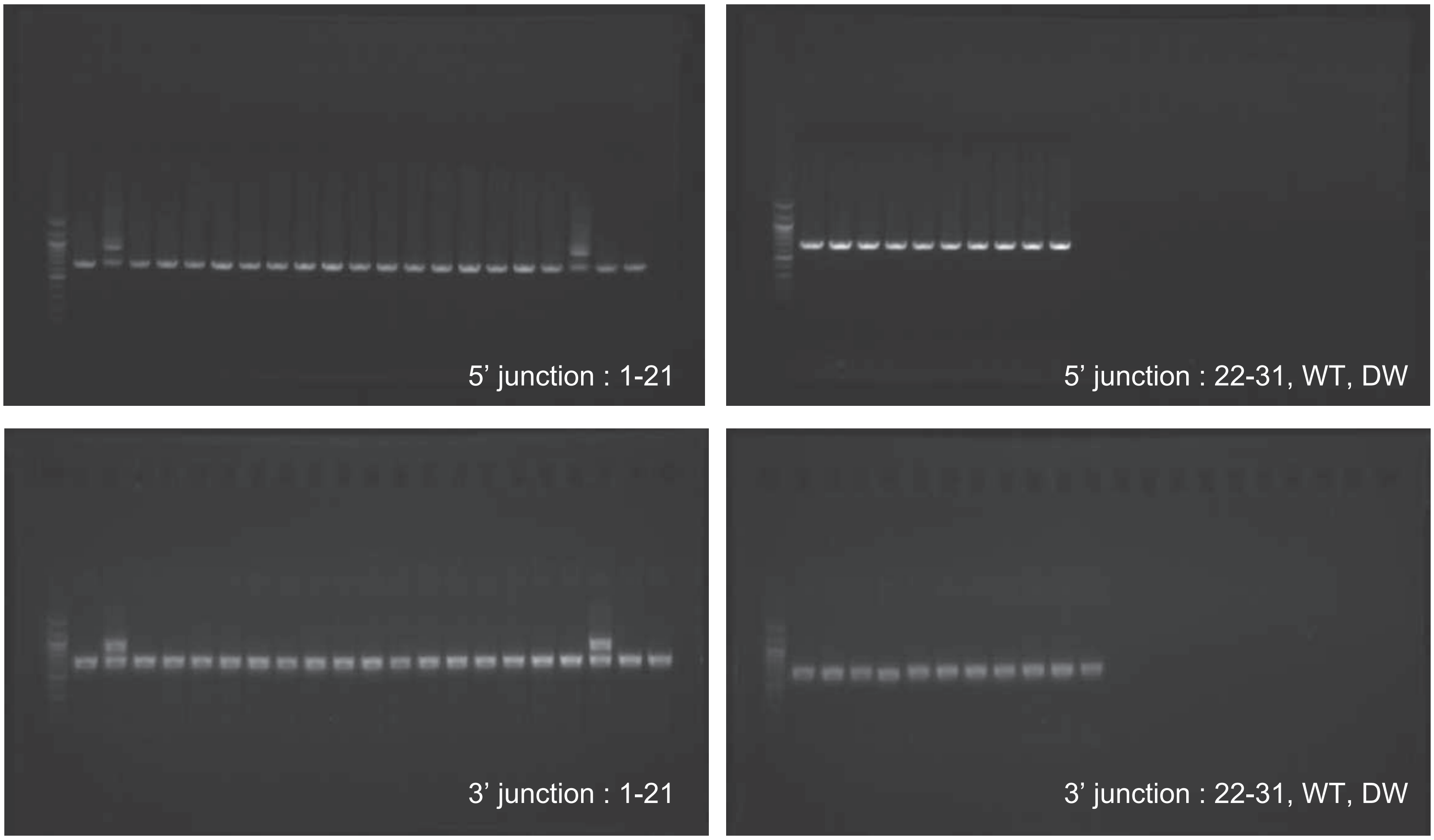

Related to supplementary figure S4 (B)

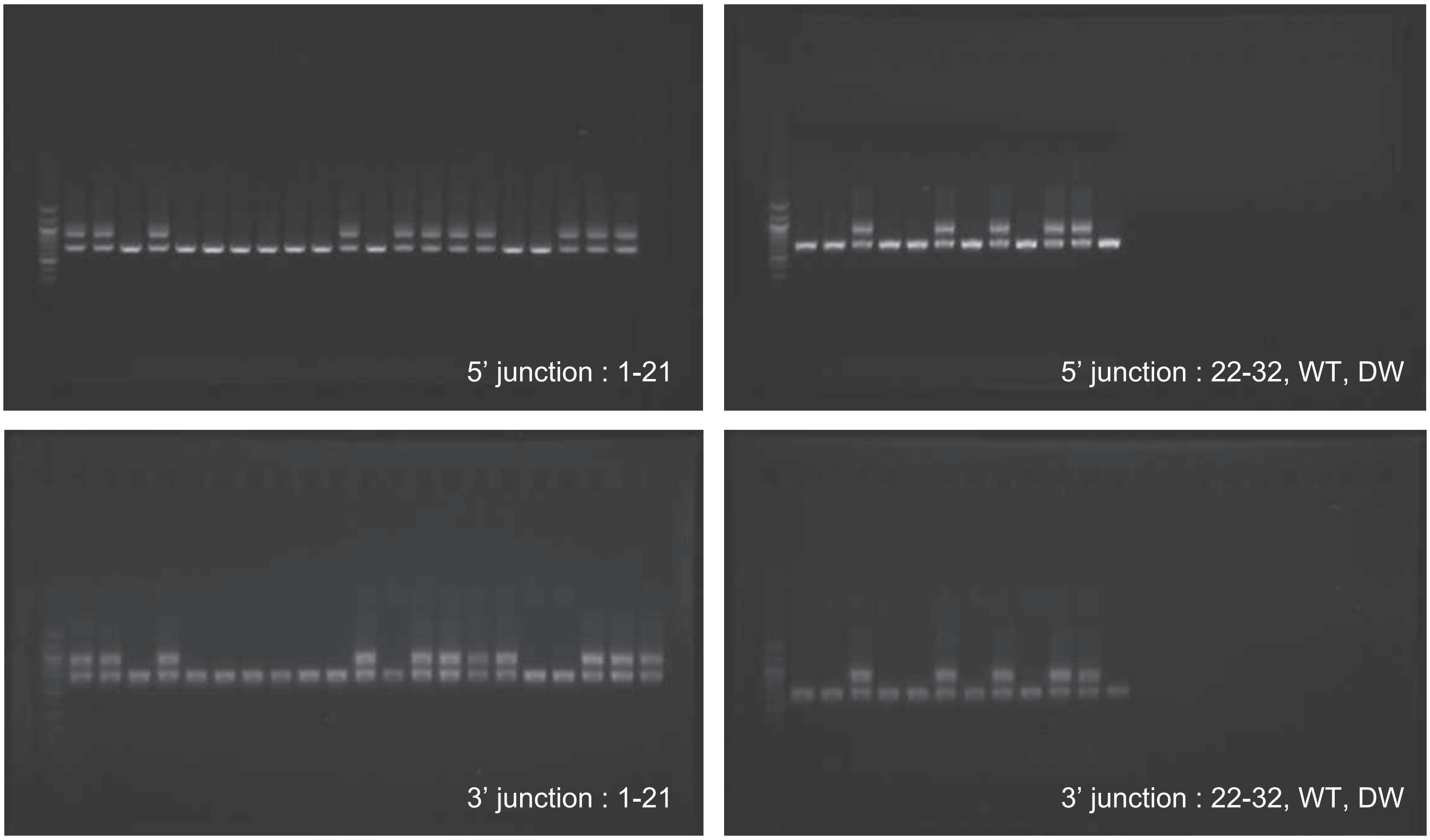

Related to supplementary figure S5 (A) (B)

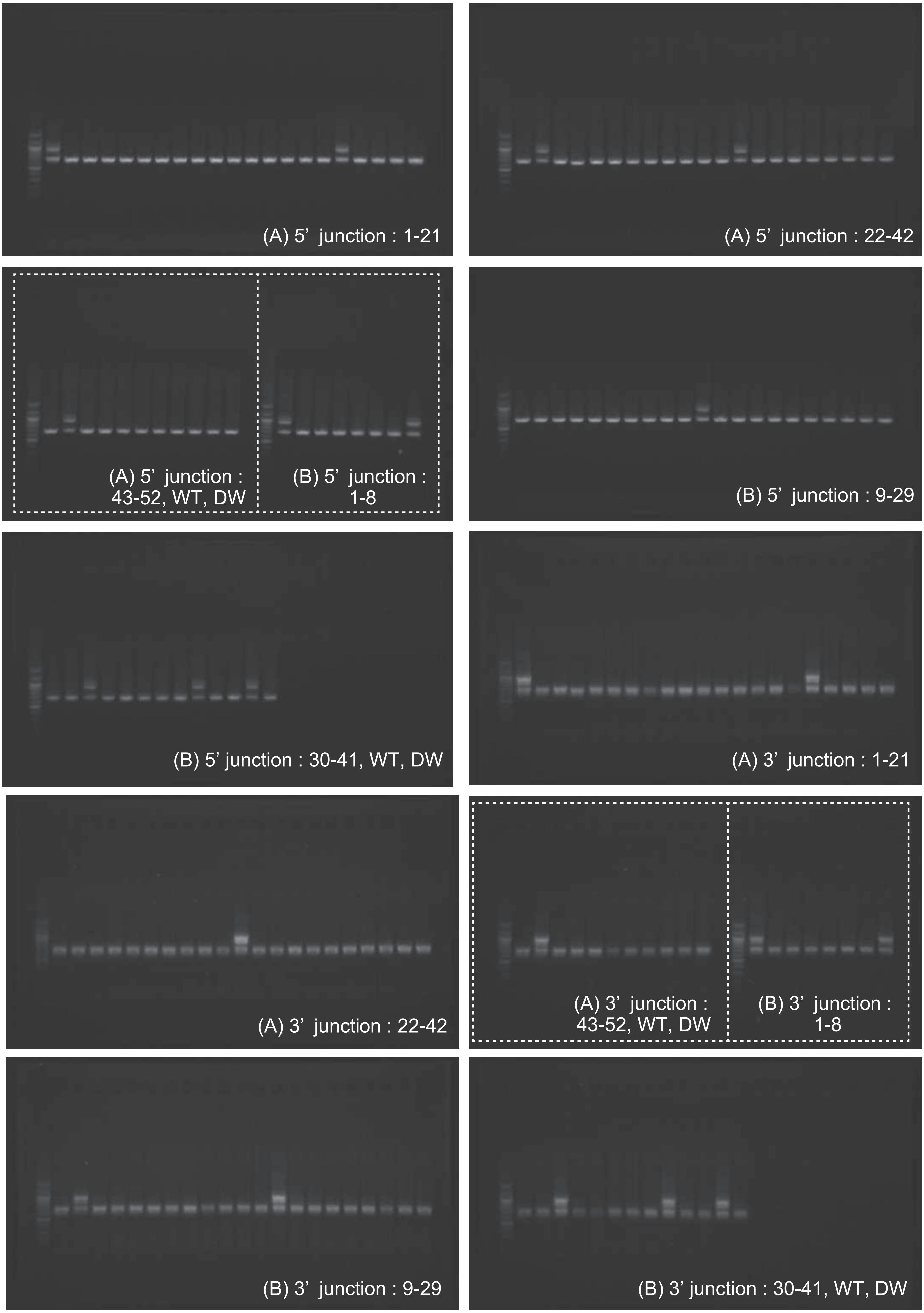

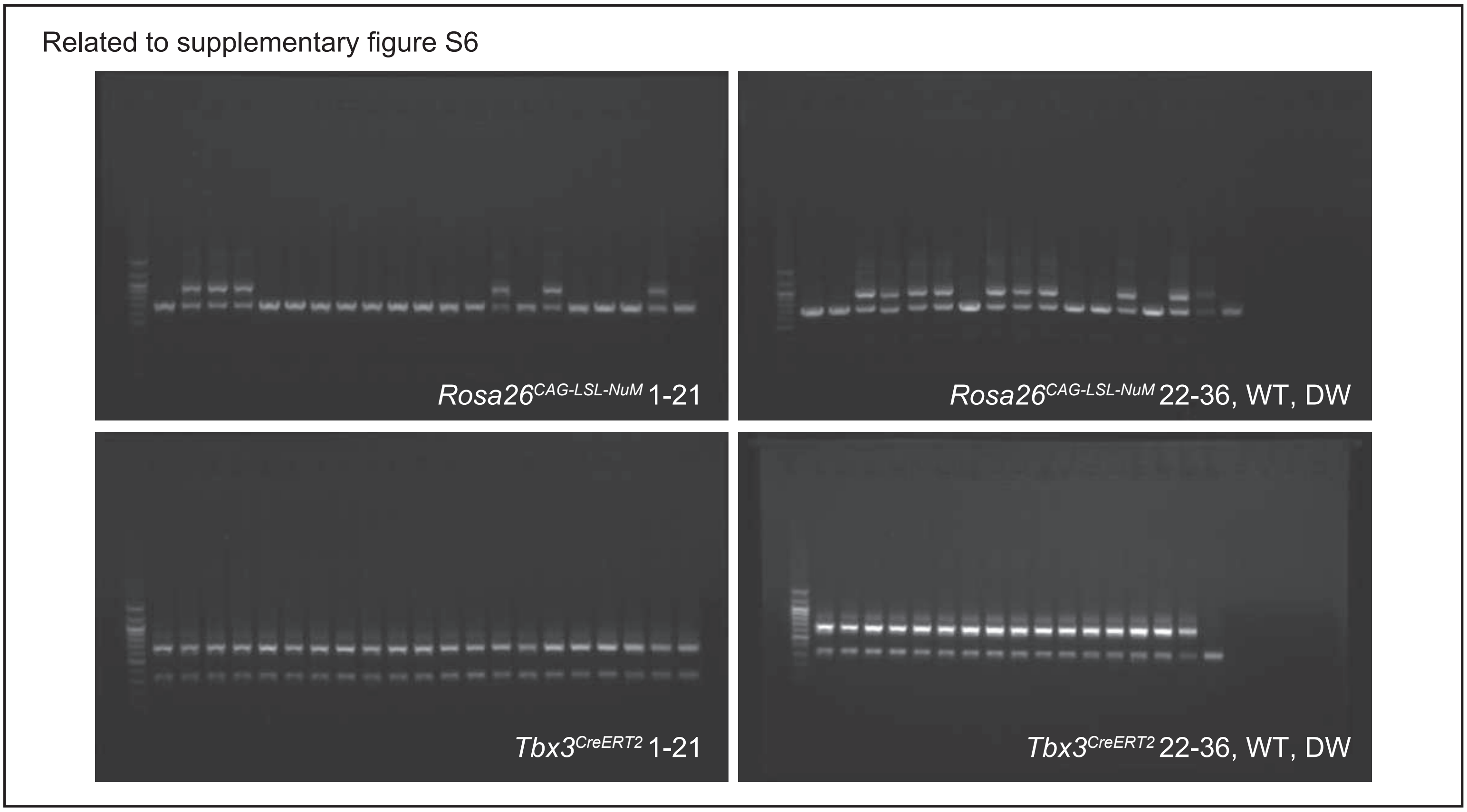

Supplementary Figure S10. Full-length, unprocessed Scan for PCR gel.
